# Preliminary results of the European multicentric phase III trial regarding sirolimus in slow-flow vascular malformations

**DOI:** 10.1172/jci.insight.173095

**Published:** 2023-11-08

**Authors:** Emmanuel Seront, An Van Damme, Catherine Legrand, Annouk Bisdorff-Bresson, Philippe Orcel, Thomas Funck-Brentano, Marie-Antoinette Sevestre, Anne Dompmartin, Isabelle Quere, Pascal Brouillard, Nicole Revencu, Martina De Bortoli, Frank Hammer, Philippe Clapuyt, Dana Dumitriu, Miikka Vikkula, Laurence M. Boon

**Affiliations:** 1 Center for Vascular Anomalies, Cliniques universitaires Saint-Luc, University of Louvain, VASCERN VASCA European Reference Centre, Brussels, Belgium.; 2 Institut Roi Albert II, Department of Medical Oncology, and; 3 Institut Roi Albert II, Department of Pediatric Hematology & Oncology, Cliniques universitaires Saint-Luc, University of Louvain, Brussels, Belgium.; 4 ISBA/LIDAM, UCLouvain, Louvain-la-Neuve, Belgium.; 5 Neuroradiology Department of Pr Houdart Lariboisière Hospital, Center of vascular anomalies clinic VASCERN VASCA European Reference Centre, Paris, France.; 6 Department of Rheumatology – DMU Locomotion, AP-HP Nord – University of Paris and INSERM U1132 BIOSCAR, Paris, France, Paris, France.; 7 Vascular Medicine Department, CHU Amiens-Picardie, Amiens, France.; 8 Department of Dermatology, CHU Université Caen Normandie, Caen, France.; 9 IDESP, Univeristy of Montpellier – INSERM, CHU Montpellier, CRMR FAVA-Multi, Montpellier, France.; 10 Human Molecular Genetics, de Duve Institute, University of Louvain, Brussels, Belgium.; 11 Centre for Human Genetics, Cliniques universitaires Saint-Luc, University of Louvain, Brussels, Belgium.; 12 Division of Interventional Radiology, and; 13 Department of Pediatric Radiology, Cliniques universitaires Saint-Luc, University of Louvain, Brussels, Belgium.; 14 WELBIO department, WEL Research Institute, Wavre, Belgium.; 15 Division of Plastic Surgery, Cliniques universitaires Saint-Luc, University of Louvain, Brussels, Belgium.

**Keywords:** Angiogenesis, Clinical Trials, Genetic diseases, Lymphomas

## Abstract

**BACKGROUND:**

Slow-flow vascular malformations frequently harbor activating mutations in the PI3K/AKT/mTOR cascade. Phase II trials pinpointed sirolimus effectiveness as a drug therapy. Efficacy and safety of sirolimus thus need to be evaluated in large prospective phase III trials.

**METHODS:**

The Vascular Anomaly-Sirolimus-Europe (VASE) trial, initiated in 2016, is a large multicentric prospective phase III trial (EudraCT 2015-001703-32), which evaluates efficacy and safety of sirolimus for 2 years in pediatric and adult patients with symptomatic slow-flow vascular malformations. In this interim analysis, we studied all patients enrolled up to October 2021 who received sirolimus for 12 or more months or who prematurely stopped the treatment.

**RESULTS:**

Thirty-one pediatric and 101 adult patients were included in this analysis; 107 completed 12 or more months of sirolimus, including 61 who were treated for the whole 2-year period. Sirolimus resulted in a clinical improvement in 85% of patients. The efficacy appeared within the first month for the majority of them. Grade 3–4 adverse events were observed in 24 (18%) patients; all resolved after treatment interruption/arrest. Sirolimus increased feasibility of surgery or sclerotherapy in 20 (15%) patients initially deemed unsuitable for intervention. Among the 61 patients who completed the 2-year treatment, 33 (54%) reported a recurrence of symptoms after a median follow-up of 13 months after sirolimus arrest. While there was no difference in efficacy, clinical improvement was faster but subsided more rapidly in *PIK3CA*-mutated (*n* = 24*)* compared with *TIE2*-mutated (*n* = 19) patients.

**CONCLUSION:**

Sirolimus has a high efficacy and good tolerance in treatment of slow-flow vascular malformations in children and adults.

**TRIAL REGISTRATION:**

ClinicalTrials.gov NCT02638389 and EudraCT 2015-001703-32.

**FUNDING:**

The Fonds de la Recherche Scientifique (FNRS grants T.0247.19, P.C005.22, T.0146.16, and P.C013.20), the Fund Generet managed by the King Baudouin Foundation (grant 2018-J1810250-211305), the Walloon Region through the FRFS-WELBIO strategic research programme (WELBIO-CR-2019C-06), the MSCA-ITN network V.A. Cure no. 814316, the Leducq Foundation Networks of Excellence Program grant “ReVAMP” (LFCR grant 21CVD03), the European Union’s Horizon 2020 research and innovation programme under grant agreement no. 874708 (Theralymph), the Swiss National Science Foundation under the Sinergia project no. CRSII5_193694, and a Pierre M. fellowship.

## Introduction

Vascular malformations result from defects in vasculogenesis, angiogenesis, and lymphangiogenesis. They are divided based on blood flow velocity into slow-flow malformations (capillary, CM; venous, VM; lymphatic, LM), fast-flow malformations (arteriovenous malformations), and combined malformations. VMs and LMs are the most common slow-flow malformations. They can be well localized or extensive. VMs can affect any tissue or organ; they are easily detected on the skin. They are usually blue-colored, soft, and compressible, and intralesional phleboliths are common. Histologically, they are characterized by enlarged venous-like channels with relative lack of smooth muscle cells. LMs consist of multiple abnormal lymph-filled cysts, which are covered by a single layer of lymphatic endothelial cells. Two subtypes are recognized: LMs with large cysts (macrocystic) and lesions that infiltrate tissues (microcystic) (1–3). These slow-flow vascular malformations are usually present at birth and grow with the child (1–3). Expansion often reduces quality of life (QoL), with chronic pain, deformation, infections, oozing, bleeding, coagulopathies, functional limitations, and even sometimes death. Therapeutic options, surgical resection, and sclerotherapy are rarely curative, often unfeasible, and/or poorly effective (4–6).

Excessive activation of the phosphoinositol-3-kinase (PI3K)/protein kinase B (AKT)/mammalian target of rapamycin (mTOR) cascade is frequently observed in slow-flow vascular malformations, resulting in locally uncontrolled development of blood vessels (7–19). Up to 60% of VMs have an activating mutation in the *TEK* gene encoding TIE2, a tyrosine kinase receptor on the surface of vascular endothelial cells, leading to activation of PI3K. Another 20% of VMs have an activating mutation in the *PIK3CA* gene encoding the PI3K catalytic subunit (10–16). Activating mutations of *PIK3CA* are also observed in up to 75% of LMs, as well as combined malformations, such as Klippel-Trenaunay syndrome (KTS); congenital lipomatous overgrowth, vascular malformations, epidermal nevis, spinal/skeletal anomalies/scoliosis (CLOVES); and PIK3CA-related overgrowth spectrum (PROS) syndromes (17, 18).

The efficacy of the mTOR inhibitor sirolimus for the treatment of slow-flow vascular malformations was prospectively evaluated in a small number of phase II trials, all having inherent limitations in terms of patient number, patient population (mostly children, vascular malformation types, absence of genomic studies), and design (undefined treatment time duration versus 12 months) (15, 20–23). In 2016, we initiated a large multicentric prospective phase III trial to evaluate these points for pediatric and adult patients with slow-flow vascular malformations. We present here the interim analysis of patients who had 12 or more months of follow-up after treatment onset or who prematurely stopped the treatment.

## Results

### Patient population.

**Figure 1:**
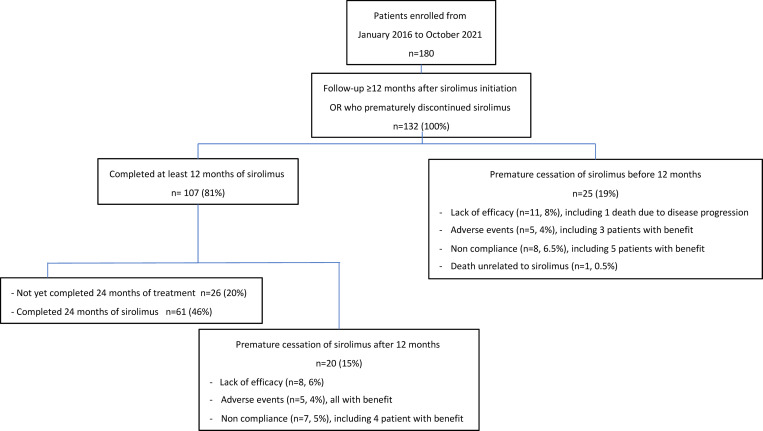
CONSORT diagram.

One hundred thirty-two patients (Figure 1: CONSORT diagram) were included (31 patients <19 years and 101 adult patients), with a median age of 30 years (3 months to 73 years) (Table 1 and Supplemental Table 1; supplemental material available online with this article; https://doi.org/10.1172/jci.insight.173095DS1). One hundred and thirteen patients (85.6%) reported pain at baseline, including 101 with continuous daily pain, 104 who had at least one pain exacerbation crisis/month, and 106 patients (80%) with a functional limitation (Supplemental Figure 1).

### General efficacy of sirolimus within the first year.

Among the 132 patients, 107 had completed 12 or more months of sirolimus treatment, 61 of whom having terminated the 2-year treatment. Among the 132 patients, 112 (85%) had a clinical improvement on sirolimus, whereas 10 patients (7.5%) never reported an improvement during the first year. Another 10 patients (7.5%) did not experience sufficient benefit within the first year.

**Figure 2:**
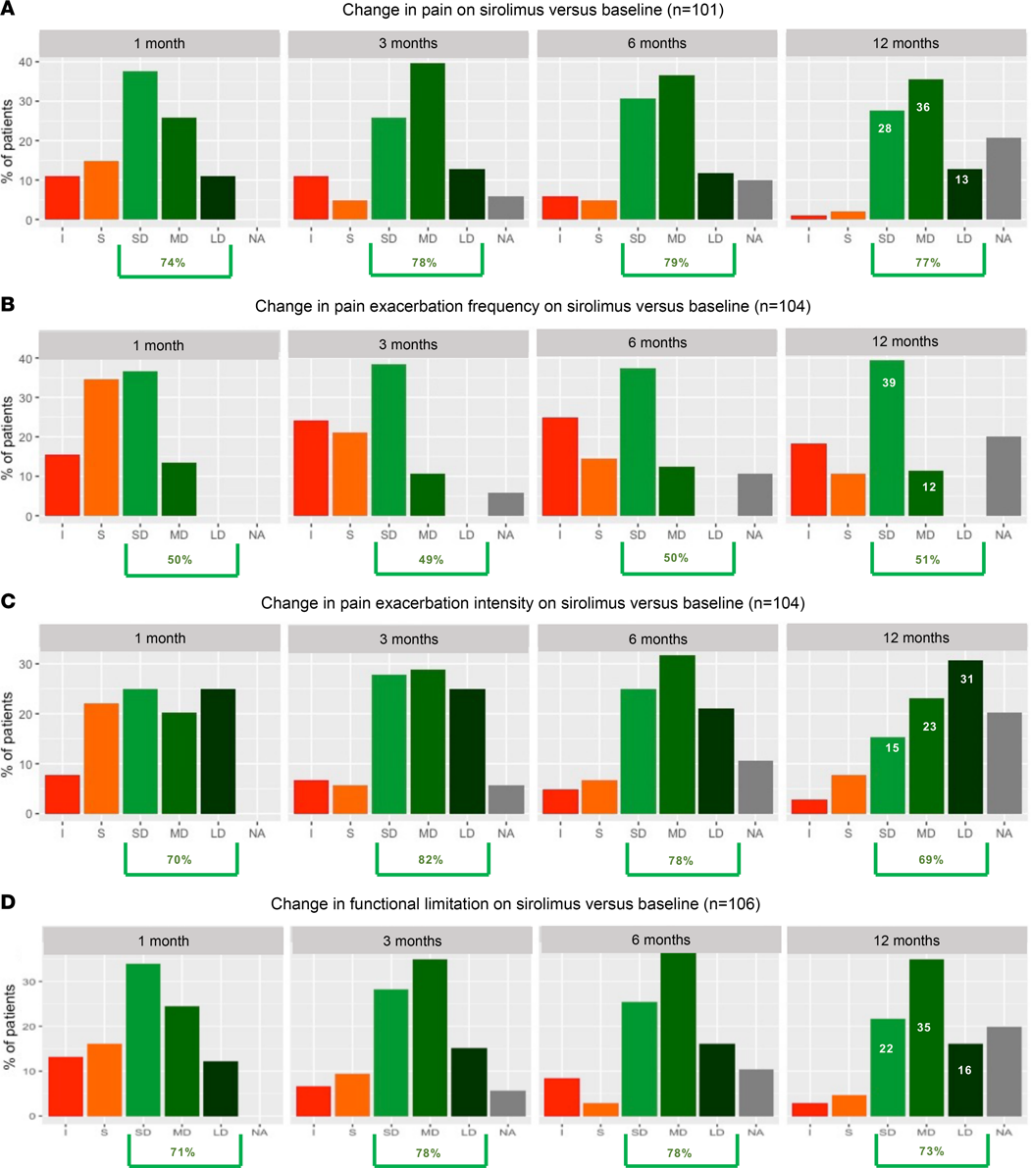
Evolution of symptoms on sirolimus. Change in pain (**A**), pain exacerbation frequency (**B**), pain exacerbation intensity (**C**), and functional limitation (**D**) from baseline to 1, 3, 6, and 12 months after start of sirolimus treatment. Change is expressed for pain (**A**) and pain exacerbation intensity (**C**), as increase (I), stabilization (S), small decrease (SD, 1 to 3 points), medium decrease (MD, 4 to 6 points), and large decrease (LD, 7 to 10 points) in pain score. For pain exacerbation frequency (**B**), the change is expressed as I, S, SD of 1 to 3 crises per month, MD of 4 to 6 crises per month, and LD of 7 to 10 crises per month. For functional limitation (**D**), change is expressed as I, S, SD (1 to 3 points), MD (4 to 6 points), and LD (7 to 10 points) in functional limitation score. Patients with missing information or out of study were grouped under “No available information” (NA).

At the 12-month time point, 77% of the patients with continuous daily pain (*n* = 101) had a reduction in pain intensity (28% with small decrease, 36% with medium decrease, and 13% with large decrease) (Figure 2A). The first improvement in pain score was reported at 1 month for 74% of the patients. Moreover, 51% and 69% of patients with baseline pain exacerbations (*n* = 104) had a reduction in pain exacerbation frequency (39% with a small decrease and 12% with a medium decrease) and in pain exacerbation intensity (15% with a small decrease, 23% with a medium decrease, and 31% with a large decrease), respectively (Figure 2, B and C). This reduction was observed at 1 month in 50% and 70% of patients, respectively.

Some patients reported an initial increase in pain compared with baseline within the first 3 months: transitory increase in continuous pain (11% and 6% of patients with and without baseline pain, respectively), increased frequency of pain exacerbations (24% and 7% of patients with and without baseline pain exacerbation), and an increase in pain exacerbation intensity (7% and 7% of patients with and without baseline pain exacerbation) (Figure 2).

At the 12-month time point, 73% of patients with baseline functional limitation (*n* = 106) reported an improvement. However, sirolimus treatment was associated with increased functional limitation within the first month in 13% and 19% of patients with or without baseline functional limitation, respectively. This proportion was reduced to under 10% with the continuation of sirolimus (Figure 2D).

### Reasons for premature sirolimus discontinuation.

Globally, 45 patients stopped sirolimus before the planned duration of 2 years (including 25 before the 12 months time point). Nineteen patients (14%) stopped because of lack of efficacy, including 1 death due to progression of a severe generalized lymphatic anomaly (GLA). Another patient died in the context of pericardial drainage not thought to be related to sirolimus. Ten patients (8%) stopped due to adverse events (AEs) despite a benefit observed in 8 of them. Fifteen patients (11.5%) stopped for noncompliance, despite a benefit observed in 9 of them.

### Safety of sirolimus.

Sirolimus induced AEs in 127 patients, resulting in a toxicity rate of 96% (Table 2). These toxicities were most commonly mild and easily manageable, with a majority of grade 1 and 2. Twenty-four patients (18%), 10 children and 14 adults, developed a grade 3 or 4 toxicity, all resolving after treatment was interrupted or arrested.

The most common toxicities were asthenia (70% grade 1–2, 4% grade 3–4), mucositis (66% grade 1–2, 8% grade 3–4), diarrhea (40% grade 1–2, 2% grade 3–4), headache (25% grade 1–2, 5% grade 3–4), cutaneous rash (31% grade 1–2, 1% grade 3–4), and gastroesophageal reflux (21% grade 1–2, 0% grade 3–4) (Table 2). AEs resulted in definitive discontinuation of sirolimus in 10 patients (8%), despite a benefit for 8 of them. During the first year of treatment, sirolimus was interrupted in 28 patients due to diarrhea (grade 2, *n* = 3; grade 3, *n* = 2), asthenia (grade 3, *n* = 4), mucositis (*n* = 9), sirolimus-unrelated reasons, or seasonal infections with fever (*n* = 10 children). Median duration of interruptions was 7 days (range 2–108 days, IQR 4–17 days).

Six patients (5%) developed a serious AE during the treatment period.

A 52-year-old woman with an orbital VM was diagnosed with an early-stage pancreatic cancer that was diagnosed on follow-up laboratory analysis 15 months after initiation of sirolimus. Sirolimus was stopped and surgery resulted in a complete remission after 36 months.

A 40-year-old woman with an extensive VM of the lower limb had a pulmonary embolism 2 months after sirolimus onset; it resolved after therapeutic low molecular weight heparin. Sirolimus was stopped.

A 48-year-old woman with a lower limb capillaro-venous malformation (CVM) and with preexisting, but controlled cardiovascular risk factors (hypertension, hypercholesterolemia, and familial cardiac ischemic event) had a transient ischemic cerebral stroke 1 year after initiation of sirolimus. She completely recovered after a few days.

A 46-year-old woman with a thoracic VM was diagnosed with breast cancer after 9 months of sirolimus. Sirolimus was continued during neoadjuvant chemotherapy, surgery, and radiotherapy, due to clinical benefit.

A 61-year-old patient with advanced GLA died due to progression of the preexisting pleural effusion and subsequent cardiac failure, despite a 6-month sirolimus treatment.

A 23-month-old boy was treated with sirolimus for pleuro-pericardial effusions and exudative enteropathy with a rapid clinical response (withdrawal of assisted ventilation, a decrease in pleural drainage, and decrease in parenteral nutrition). Twelve months after sirolimus initiation, pericardial drainage was performed in another institution for an asymptomatic pericardial effusion detected on cardiac ultrasonography during an elective consultation. The procedure resulted in massive bleeding and death.

### Sirolimus serum level and efficacy.

Serum trough levels of sirolimus were assessed after 1 month of treatment, allowing adaptation of sirolimus doses to reach a serum level ranging between 10 and 15 ng/mL. Among the 18 patients who had transient worsening at 1 month compared with baseline and with available serum level of sirolimus, 9 were below 10 ng/mL and the other 9 were at 10 ng/mL or higher. The serum levels varied between 4.3 and 15.2 ng/mL. In the 69 patients who presented an improvement at 1 month compared with baseline and with available serum level (*n* = 67), 45 were below 10 ng/mL and 22 were at 10 ng/mL or higher. The serum levels varied between 1.8 and 23.4 ng/mL.

### Beyond the 2-year treatment with sirolimus.

**Figure 3:**
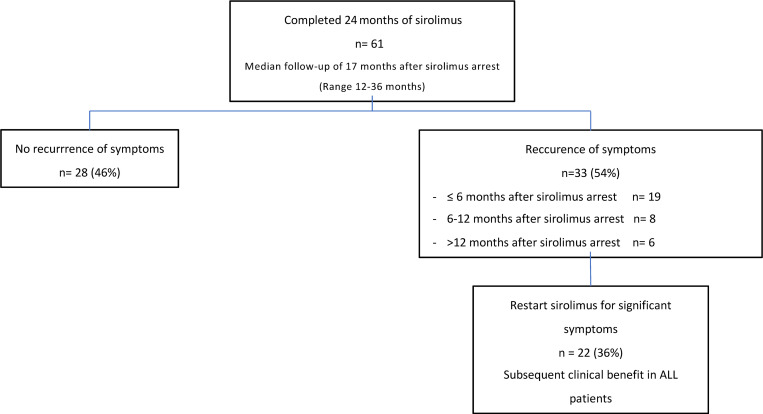
Evolution of patients after the 2-year sirolimus treatment.

Based on the 107 patients who completed at least 1 year of treatment, sirolimus resulted in feasibility of surgery in 14 patients (7 during the second year of sirolimus treatment and 7 after the 2-year sirolimus treatment) and sclerotherapy in 4 patients (all after the 2-year sirolimus treatment). While deemed feasible in an additional 2 patients after 2 years of treatment, surgery was refused, as clinical benefit was already met. Among the 61 patients who completed the 2-year sirolimus treatment, and after a median follow-up after sirolimus cessation of 17 months (ranging from 12 months to 36 months), 33 patients (54%) reported symptom recurrence (pain and/or functional limitation): 19 of them within 6 months after discontinuation of sirolimus, 8 between 6 and 12 months, and 6 after 12 months. Twenty-two patients (36%) restarted sirolimus (at the same dosage that previously resulted in the best clinical effect), after a median of 6 months (range 1–18 months) from discontinuation of treatment, due to marked symptom resurgence, all with subsequent clinical benefit (Figure 3). The remaining 11 patients did not want to restart sirolimus, as their recurrence was mild. During the period of sirolimus administration in the Vascular Anomaly-Sirolimus-Europe (VASE) trial, pregnancies were contraindicated. However, this was lifted after cessation of treatment. One female patient experienced 2 noncomplicated pregnancies within 3 years after sirolimus arrest and 1 male patient became a father within 2 years after sirolimus arrest; all children develop normally. One patient is currently pregnant (6-month gestational age) less than 2 years after sirolimus arrest, with normal fetal development.

### Correlation between genotype and sirolimus efficacy.

In an exploratory way, genetic testing was planned to be performed in all patients for whom tissue samples were available. Genetic results were procured for 52 patients (Supplemental Table 1); 24 (46%) had a somatic mutation in *PIK3CA* and 19 (37%) in *TIE2*. Other mutated genes included *PTEN* (*n* = 3), *GNAQ* (*n* = 4), glomulin (*GLMN*) (*n* = 1), and *GNA11* (*n* = 1). While *TIE2* mutations were observed only in VMs, *PIK3CA* mutations were observed also in LMs and combined vascular malformations: CVM, capillary malformation with dilated veins (CMDV), capillaro-lymphatico-venous malformation (CLVM), CLOVES, and KTS.

A sustained benefit of sirolimus was observed in 84% of *TIE2*-mutated and 83% of the *PIK3CA*-mutated patients, as well as in the 3 *PTEN*-, 2 *GNAQ*-, the 1 *GLMN*-, and the 1 *GNA11*-mutated patient. The rapidity and intensity of pain response between *PIK3CA*- and *TIE2*-mutated patients varied; 67% of the *PIK3CA*-mutated patients experienced improvement in the first month of treatment compared with 26% of the *TIE2*-mutated patients. Moreover, 63% of *PIK3CA*-mutated patients experienced improvement in functional limitation during the first month of treatment compared with 37% of *TIE2*-mutated patients.

Among the 61 patients who completed the 2-year treatment, a somatic mutation was known for 30 (49%); mutations were in *PIK3CA* (*n* = 15), *TIE2* (*n* = 12), *GNAQ* (*n* = 1), *GLMN* (*n* = 1), and *PTEN* (*n* = 1). Vascular malformation–related worsening of the status after sirolimus interruption was experienced by 9 patients with a *PIK3CA* mutation (at 3 months [*n* = 8] or 6 months [*n* = 1]), by 6 patients with a *TIE2* mutation (at 9 months [*n* = 5] or 18 months [*n* = 1]), and by 1 patient with a *PTEN* mutation (at 3 months).

## Discussion

To date, this is the largest prospective clinical phase III trial evaluating the efficacy of a 2-year sirolimus treatment in slow-flow vascular malformations. In this mid-term study evaluating 132 patients, a clinical benefit rate of 85% was observed. This corroborates the results of the phase II trials (15, 20–22).

Sirolimus had a relatively rapid effect on slow-flow vascular malformations; 50% of patients reported improvement of their symptoms within the first month of treatment. The proportion of patients with benefit increased with time, even if a transient deterioration in symptomatology was detected in up to 20% of patients during the first month of treatment. This highlights the importance of avoiding premature discontinuation of sirolimus and of informing patients that symptoms can transiently worsen at the beginning of the treatment. Sirolimus allowed surgery or sclerotherapy in patients who were initially deemed ineligible for such therapies. This underscores the essential role sirolimus may have if introduced before invasive therapies.

The range of serum sirolimus levels was very large in this trial, ranging from 4.3 to 15.2 ng/mL. This could be explained by the fact that patients with a clinical benefit on sirolimus, whatever the trough level, were allowed to continue with the same dose; some of them had a low serum level of sirolimus. This reflects the interindividual variation in sensitivity to sirolimus. Treating very young children may require a close follow-up with more frequent clinical visits during the first weeks, in order to avoid toxicity, as sirolimus levels may change rapidly in a short period of time.

The toxicity profile was consistent with previous reports; 18% of patients had a grade 3–4 toxicity, mainly asthenia, mucositis, headache, and skin rash. A higher frequency of grade 3–4 mucositis was observed in children, while adults had more frequently grade 3–4 asthenia. No hematological toxicity was observed in children or adults. Toxicity resulted in definitive discontinuation in 7 patients. The absence of hematological toxicity was consistent with our previous experience in our clinical pilot trial and our phase II study (15, 20). However, close collaboration with pediatric hematological oncologists remains mandatory, as 27% of grade 3 bone marrow toxicity was observed in a population of children and young adults with LM (*n* = 60; median age 8 years, ranging from 21 days to 28 years) (23). The reason for this discrepancy is unclear, but could be related to the vascular disease characteristics in the cohorts or to the fact that we based sirolimus dosage on clinical benefit rather than on serum trough levels (23).

Based on our experience with phase IB and II studies (15, 20), and based on the fact that no *Pneumocystis* infection has been related to sirolimus monotherapy in the vascular field (8), we did not systematically offer *Pneumocystis* prevention to our patients in the VASE trial. During this midterm analysis, no *Pneumocystis* infection was detected, underscoring that *Pneumocystis* prevention is not systematically needed. However, a close collaboration with pediatric hematological oncologists remains essential and prophylactic antibiotics may be considered on a case-by-case basis in patients who develop dose-dependent lymphopenia or neutropenia, for children with underlying lung-dysfunction, a poor general condition or other comorbidities, and in very young children (<3 months of age) (8). This may also be given in case of parental preference.

Two patients developed cancer during sirolimus treatment, yet these malignancies were unlikely to be related to sirolimus. First, these malignancies appeared too early after the initiation of sirolimus to have a relationship with sirolimus itself. Secondly, the association between sirolimus and cancer remains unclear. Even if we do not have specific studies evaluating risk of cancer occurring during sirolimus monotherapy, the risk of developing cancer in graft-recipient patients on sirolimus treatment seems lower than that observed on a calcineurin inhibitor (24). Third, sirolimus is known to have anticancer effects and it is approved for treatment of renal, breast, and neuroendocrine cancers (25). As 1 patient developed transient ischemic cerebral stroke, even if unlikely related to sirolimus, we recommend close and regular control of cardiovascular parameters such as body mass index, arterial tension, and cholesterol profile on sirolimus.

Influence of sirolimus on fertility has been a concern, mostly based on studies on organ transplantation patients (26). Yet, it seems to have limited, if any, effect in monotherapy. We have observed 3 pregnancies during the follow-up period after cessation of sirolimus administration. With the full follow-up period of the complete VASE study, we will hopefully obtain more data.

**Figure 4:**
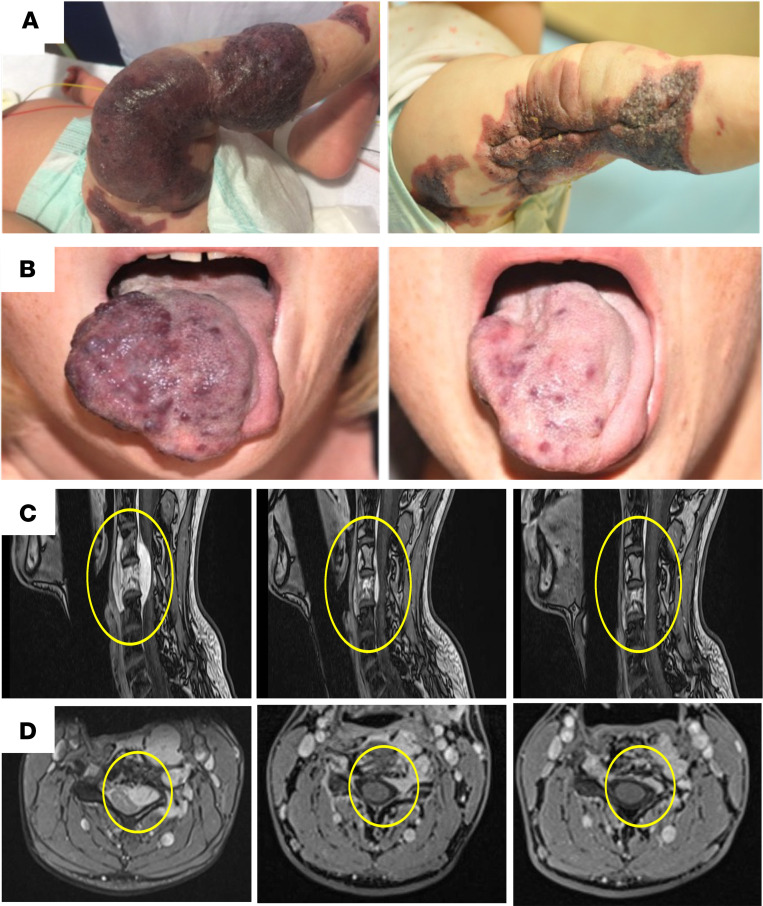
Representative clinical and radiological evolution on sirolimus. (**A**) Evolution of malformations before (left) and after sirolimus treatment (right). Evolution after 3 months of sirolimus treatment of an extensive capillary venous malformation of the lower extremity responsible for daily bleeding and severe consumptive coagulopathy with low fibrinogen and high D-dimer (3-month-old girl). Within 1 month of sirolimus treatment, daily bleeding ceased; surgical resection became possible after 4 months of sirolimus treatment. (**B**) Evolution after 3 months of sirolimus treatment of a tongue venous malformation (20-year-old girl). (**C** and **D**) MRI axial (**C**) and sagittal view (**D**) showing the evolution of a venous malformation of the fourth cervical body that caused cervico-brachialgia in a 34-year-old man (left). Two-year sirolimus treatment showing regression of the malformation and restoration of the anatomy of the medullary canal (middle). He is currently treatment free 2 years after sirolimus arrest (right).

Only 36% of the patients who completed the 2-year sirolimus treatment had to restart sirolimus, suggesting that the majority of patients do not need to remain on sirolimus indefinitely (Figure 4). *PIK3CA* mutations seem to be associated with a more rapid response to sirolimus treatment compared with *TIE2* mutations, but also with a more rapid resurgence of symptoms after interruption of sirolimus treatment. This finding needs confirmation, which should come when the full VASE study cohort can be analyzed.

The limitations of the current study include the small number of children, the relative heterogeneity of the patient population, the small number of patients in some diagnostic groups, and the low number of genetic analyses. Yet, this phase III prospective trial underscores the important role that sirolimus treatment can play in the multimodal management of slow-flow vascular malformations.

## Methods

### Protocol.

This is a nonrandomized prospective trial in which all patients receive sirolimus, with their previous long-term clinical history used as their own control. The trial was registered at ClinicalTrials.gov (NCT02638389) and EudraCT (2015-001703-32) and started in January 2016.

### Patient population.

To be eligible for the VASE trial, patients had to have an LM, VM, or complex slow-flow vascular malformation that was symptomatic (pain, functional limitation, decreased QoL, recurrent infections, oozing, and/or bleeding), refractory to medical treatment (pain killers, low molecular weight heparin, or compression garments), and deemed ineligible for sclerotherapy and/or surgery. Eligible patients were 3 or more months old, had adequate hepatic (bilirubin, aspartate amino transferase [ASAT], alanine amino transferase [ALAT]), medullary (neutrophils ≥1500/mm³, hemoglobin ≥8.0 mg/dL, and platelets ≥50,000/mm³) and renal function (clearance ≥70 mL/min/1.73 m²), a Karnofsky performance status of 50 or higher, and no concurrent use of cytochrome P450 3A4 enzyme inducers or inhibitors.

Exclusion criteria included patients with severe concurrent and/or uncontrolled disease (impaired cardiac function or clinically important cardiac disease, impairment of gastrointestinal absorption, history of a primary malignancy within the past 5 years, immunocompromised state, such as for those infected with HIV), and pregnant or lactating women. Possible adverse effects, including that of eventual fertility problems, were discussed with all patients before enrolment. Patients could not receive surgery and/or sclerotherapy within 4 weeks prior to study entry.

Malformations that involved more than 1 anatomical region (delimited as forehead, orbit, maxillary, mandibular, neck, trunk, back, abdomen, thigh, leg, foot, arm, forearm, or hand) were defined as “extensive” and those involving a muscle and/or joint and/or bone and/or internal organs as “deep.” There were 103 patients from Brussels, 12 from Paris, 10 from Amiens, 6 from Caen, and 1 from Montpellier, with 86 females (65%) and 46 males (35%). All patients were highly symptomatic and most of them (67%) had had several sessions of sclerotherapy and/or surgical resections, with continued poor QoL.

At the time of analysis (October 2022), 220 patients had been enrolled. This interim analysis focuses on patients enrolled up to October 2021, who thus have had 12 or more months of follow-up after treatment onset. This also includes patients who prematurely stopped treatment due to loss of follow-up, toxicity, or other reasons.

### Study protocol and treatment.

Sirolimus was started at a dose of 0.8 mg/m^2^ body surface, twice a day in the form of a liquid solution for children under the age of 12 years, and with a single dose of 2 mg/day in the form of a tablet for older patients. During the treatment period, study visits were performed every month for the first 3 months, and then every 3 months. After 1 month and 6 months of treatment, sirolimus blood levels were measured. Sirolimus doses could be increased in case of low serum trough levels, absence of important toxicity, and patient’s agreement. Conversely, patients with low trough levels and clinical benefit were allowed to continue on the same daily dose of sirolimus.

Fluctuating sirolimus levels can be observed in very young patients (<6 months old), requiring frequent dose adaptations, mainly during the first weeks of treatment (8, 23). Even if trough-level follow-up during the first weeks of treatment was not required per protocol, regular concentration checks were performed at the physician’s discretion, in very young children. The frequency of controls depended on trough levels and the required dose adaptations.

Grade 3–4 toxicities required an interruption of the treatment until recovery to grade 1 or higher; sirolimus could subsequently be reintroduced at the initial dose or lower dose, depending on toxicity type, grade, and patient’s preference. Biological tests (including D-dimer and fibrinogen levels) were controlled at baseline, after 1 month, and then every 6 months. Magnetic resonance imaging (MRI) (T1-weighted without gadolinium chelate injection, T2-weighted with Fat Saturation or STIR sequences in 2 orthogonal planes) was performed at baseline and then every 12 months.

Sirolimus was administered for a planned treatment duration of 2 years, with a further follow-up of 5 years and frequent visits (every 3 months for the first year and then every 6 months). Sirolimus was allowed to be reintroduced for an undefined duration in case of symptomatic relapse. Treatment was suspended in case of grade 3–4 toxicity related to medication, patient and/or parents who refused to continue, and patients who did not continue their follow-up.

### Genetic testing.

Somatic genetic analysis was performed on available fresh or archival tissues. See supplemental material for details.

### Objective of the trial.

The primary objectives were to assess the efficacy of sirolimus for the treatment of slow-flow vascular malformations and to evaluate the associated safety profile in children and adults. The efficacy assessment of sirolimus at each visit was based on the following.

Patient self-assessment of the vascular malformation-related general evolution with sirolimus compared with last visit: improvement, stability or worsening.

Evaluation of symptoms focused on pain (intensity of continuous pain and frequency/intensity of pain exacerbation), functional limitation, bleeding, oozing, and repetitive infections. The evaluation of pain (continuous and exacerbation) including the intensity based on visual analog scale (VAS), ranging from 0 (no pain) to 10 (excessive pain), and the frequency of pain exacerbations (number of crises per month). The evaluation of functional limitation (difficulties in performing any action of everyday life) based on a scale ranging from 0 (no limitation) to 10 (excessive limitation).

Size of the lesion on the basis of clinical evaluation and measurement with calipers at each consultation, and radiological comparison between baseline and 1- and 2-year MRI).

Other exploratory endpoints included the time interval between the end of treatment (after 2 years of treatment per protocol) and symptom recurrence, the efficacy of sirolimus based on vascular malformation type and/or genomic alteration, and the rate of subsequent surgery and/or sclerotherapy that became possible by sirolimus.

Health-related QoL (HRQoL) was evaluated at baseline and at each consultation based on a questionnaire modified from the RAND MOS SF-36 Survey (https://www.rand.org/health-care/surveys_tools/mos/36-item-short-form.html), and on a global self-evaluation.

Lack of benefit was defined as (a) patients who discontinued sirolimus prematurely for absence of efficacy and (b) patients who stopped sirolimus for AEs/noncompliance and without clinical benefit.

AEs were assessed according to the Common Terminology Criteria for Adverse Events v5.0 (https://ctep.cancer.gov/protocoldevelopment/electronic_applications/docs/ctcae_v5_quick_reference_5x7.pdf). If applicable, coagulopathy evolution (based on D-dimer levels) was also recorded but not considered as a response per se. For this interim analysis, we focused on the evaluation of symptoms and safety.

### Statistics.

In this interim analysis, we report descriptive statistics. Continuous variables are reported as mean and standard deviation (SD) or as median and interquartile range (IQR), as appropriate. Binary and categorical variables are reported as frequency and relative frequency (%). This interim analysis focused on efficacy and safety. All data were collected and managed using REDCap electronic data capture tools and analyzed using R software (27, 28).

In the complete VASE study, which focuses on rare diseases, all patients will be treated with the experimental treatment and the main interest is on the difference in the results measured before and after treatment. Each patient will therefore be his/her own control and comparison will be made according to paired tests (*t* test if normality can be assumed, Wilcoxon’s signed-rank test otherwise). The sample size calculation was performed based on a paired *t* test (so assuming normality), but one can consider adding 10% to the computed sample size to take into account possible departure from the normality (skewness). These sample size calculations are based on the results observed in a previous pilot study (15). The sample size calculations are based on the main objective of the study, namely the comparison of VAS, D-dimer, MRI volume, and QoL at start versus 3 months for the first 2 parameters, at start versus 12 months for MRI and at 3 months versus 12 months for QoL. This leads to 4 main comparisons and according to a Bonferroni adjustment, the type I error considered for each of these comparisons is therefore set to 1.25%. Sample size calculations aim to a power of 95%. The largest sample size is required for the comparison of QoL, with a total of 158 patients needed to have a 95% power to detect a difference of 10 units (assuming an SD of differences of 30).

The objective is also to analyze the results according to 6 diagnostic groups: (a) venous malformation, unifocal + multifocal + blue rubber bleb nevus syndrome (BRBN) + capillaro-venous malformation; (b) LM + lymphatico-venous malformation (LVM); (c) CLVM + KTS + CLOVES; (d) Gorham + GLA; (e) PTEN hamartoma tumor syndrome (PHTS); and (f) kaposiform hemangioendothelioma (KHE) + tufted angioma. Therefore, a total of 250 patients will be enrolled in the study, providing therefore a 95% power (or more) in the 3 largest groups to detect a difference of the magnitude of the one identified in the pilot study (with no adjustment per group) if the SD is the same, or even (slightly) larger (no multiplicity adjustment for the number of groups). In the smallest of these groups; the power will be of 95% to detect a difference of 2.3 (SD 2.5), 3728 (SD 4000), 75 (SD 80) for VAS, D-dimer, and MRI score, respectively, with Wilcoxon’s signed-rank test and with a type I error of 1.25%. The power will obviously be larger in the larger groups. For QoL, however, only the largest group (a) achieves a power of 95% to detect a 10% difference in QoL score between 3 months and 12 months, assuming an SD of differences of 30. The 3 smaller groups (d, e, and f) have to be grouped with other groups, or together, to achieve a reasonable power. All statistical hypothesis tests are 2-tailed unless specific otherwise. See the detailed statistical plan in supplemental material.

### Study approval.

This study was designed by investigators at Cliniques universitaires Saint-Luc, Brussels, Belgium and approved by the Biomedical Ethical Committee of the 5 hospitals involved in the study (Brussels, Belgium, and Amiens, Caen, Montpellier, and Paris, France). Each patient, or for minors, both parents of the patient, signed an informed consent after having received the patient information form adapted to patient’s age with explanations from a study nurse and the physician, and a summary explaining the procedure of the study.

### Data availability.

The data are available from the corresponding authors upon reasonable request. Supporting data used for statistical analyses are provided in the Supporting Data Values file.

Detailed aggregated study data values and the detailed study protocol are provided in the supplemental material.

## Author contributions

ES, AVD, MV, and LMB designed the research studies. All authors conducted experiments and analyzed data. ES, CL, NR, MDB, PB, MV, and LMB analyzed data. All authors contributed to writing the manuscript.

## Supplementary Material

Supplemental data

ICMJE disclosure forms

Supporting data values

## Figures and Tables

**Table 1:**
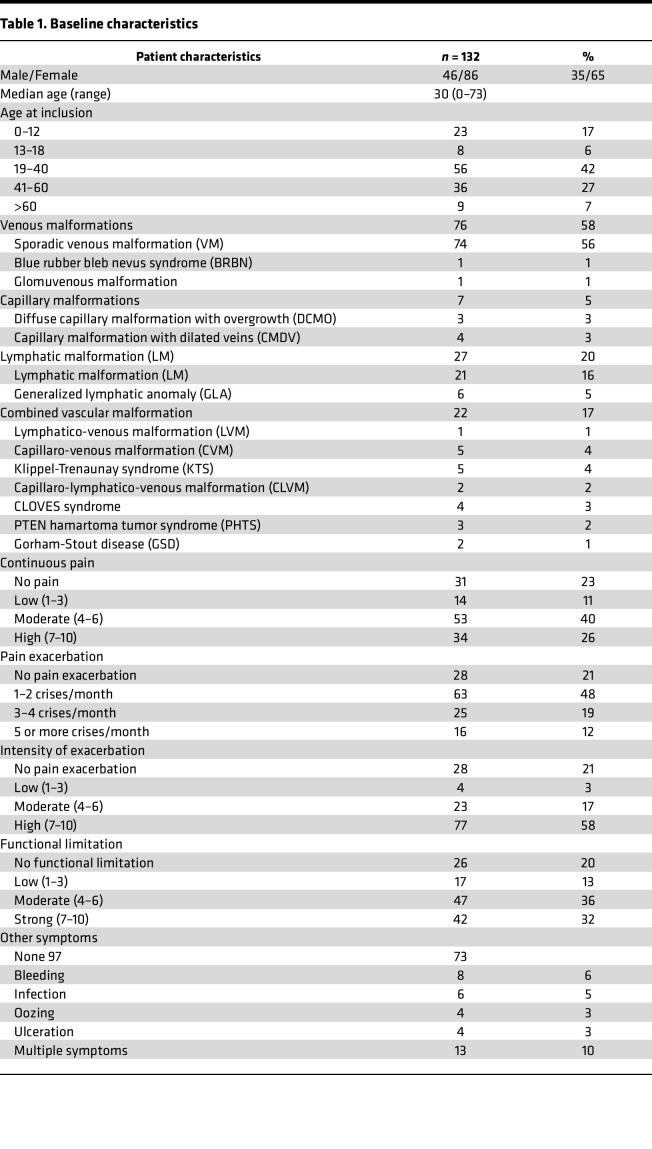
Baseline characteristics

**Table 2:**
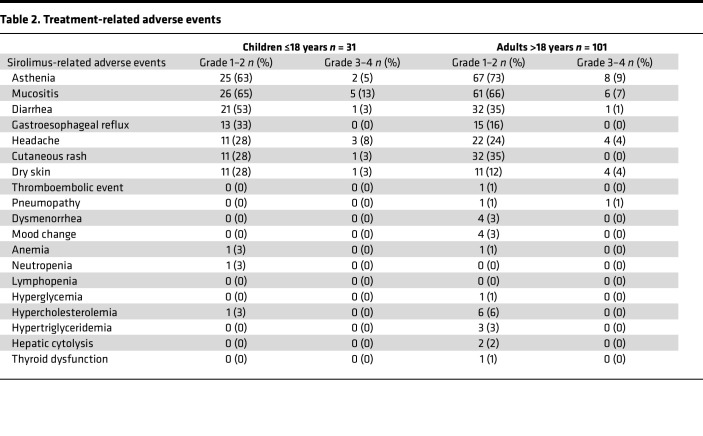
Treatment-related adverse events

## Version 1 11/08/2023

Electronic publication
