# Supplementary material for: Preliminary results of the European multicentric phase III trial regarding sirolimus in slow-flow vascular malformations
Source: JCI Insight. 2023 Nov 8;8(21):e173095. doi: 10.1172/jci.insight.173095 (PMC10721262; doi:10.1172/jci.insight.173095)
Supplement: Supplemental data [file jciinsight-8-173095-s200.pdf]

Supplementary appendix

This supplementary appendix includes

A) **Supplemental methods: Detailed statistical plan**
B) **Supplemental methods: Detailed genetic analysis**
C) **Supplementary Table (1S and 2S)**
D) **Supplementary Figure (1S)**
E) **Study protocol (Initial version 2015)**
F) **Summary of amendment made in 2018 on the initial protocol**

A) Supplemental methods: Detailed statistical plan

In this study, all patients will be treated with the experimental treatment and the main interest is on the difference in the various parameters considered as measured before and after treatment. Each patient will therefore be his/her own control and comparison will be made according to paired-tests. In case, normality can be assumed, the paired t-test will be used, otherwise statistical comparisons will rely on the Wilcoxon's signed-rank test.
The sample size is justified below acknowledging that

- There is no direct methodology to compute sample size for a non-parametric Wilcoxon's signed-rank test. It is therefore common to compute the sample size for a paired t-test (assuming approximate normality) and then to add 10% to the sample size to take skewness into account.
- Computing sample size for a paired t-test requires information on the target mean differences and on the standard deviation (SD) of differences within pairs. This information has been estimated from the data available from a previous study (Boscolo et al. JCI 2015). Since this information is only based on 6 patients, some sensitivity calculations have been performed modifying these parameters.

For the sample size calculation, it is considered that the main objective of this study is based on

- the parameters: VAS, D-dimer, MRI volume, QoL
- the comparison of results at start versus results at 3 months for VAS and D-Dimer, at start versus 12 months for MRI (since no data on measurement in between), and at 3 months versus at 12 months for QoL (as no data at start).

Sample size is computed:

- Without adjustment for multiplicity ($\alpha = 0.05$)
- With Bonferonni adjustment for multiplicity ($\alpha = 0.0125$) for the 4 parameters considered (no adjustment for the comparisons per group)

VAS – Start versus 3 months
Results observed in the Boscolo et al study are:

- mean difference of 4.6
- standard deviation of differences: 2.4

Required sample size for a 95% and <u>no multiplicity adjustment</u> ($\alpha = 0.05$) for various value of the target mean differences (Diff) et various assumed value of the standard deviation of differences:

	SD=1.5	SD=2	SD=2.5	SD=3	SD=4
Diff=3	6	8	12	16	20
Diff=4	5	6	8	10	13
Diff=5	4	5	6	7	9
Diff=6	4	4	5	6	7

NB: Need to add 10% of patients to account for skewness in the distribution of the VAS score

Required sample size for a 95% and <u>with multiplicity adjustment</u> (α = 0.0125) for various value of the target mean differences (Diff) et various assumed value of the standard deviation of differences:

	SD=1.5	SD=2	SD=2.5	SD=3	SD=4
Diff=3	8	11	16	21	27
Diff=4	6	8	11	13	17
Diff=5	5	7	8	10	12
Diff=6	5	6	7	8	10

NB: Need to add 10% of patients to account for skewness in the distribution of the VAS score

⇨ ***We can expect that a total of 15 patients would provide 95% power to detect a difference of 4 units in VAS score between start and 3 months with a Wilcoxon's signed-rank test, assuming a standard deviation of differences of 3 and a type I error of 1.25%***

D-Dimer – Start versus 3 months

Results observed in the Boscolo et al study are:

- mean difference of 4859
- standard deviation of differences: 3933

Required sample size for a 95% and <u>no multiplicity adjustment</u> (α = 0.05) for various value of the target mean differences (Diff) et various assumed value of the standard deviation of differences:

	SD=3000	SD=3500	SD=4000	SD=4500	SD=5000
Diff=4000	10	13	16	19	23
Diff=4500	8	11	13	16	19
Diff=4800	8	10	12	14	17
Diff=5000	7	9	11	13	16
Diff=5500	7	8	10	11	13

NB: Need to add 10% of patients to account for skewness in the distribution of the D-dimer score

Required sample size for a 95% and <u>with multiplicity adjustment</u> (α = 0.0125) for various value of the target mean differences (Diff) et various assumed value of the standard deviation of differences:

	SD=3000	SD=3500	SD=4000	SD=4500	SD=5000
Diff=4000	13	17	21	25	31
Diff=4500	11	14	17	21	25
Diff=4800	11	13	16	19	22
Diff=5000	10	12	15	18	21
Diff=5500	9	11	13	15	18

NB: Need to add 10% of patients to account for skewness in the distribution of the D-dimer score

⇨ ***We can expect that a total of 21 patients would provide 95% power to detect a difference of 4500 units in D-dimer score between start and 3 months with a Wilcoxon's signed-rank test, assuming a standard deviation of differences of 4500 and a type I error of 1.25%***

MRI Volume – Start versus 12 months

Results observed in the Boscolo et al study are:

- mean difference of 60.6
- standard deviation of differences: 73.80

Required sample size for a 95% and no multiplicity adjustment (α = 0.05) for various value of the target mean differences (Diff) et various assumed value of the standard deviation of differences:

	SD=50	SD=60	SD=70	SD=80	SD=90
Diff=40	23	32	42	54	68
Diff=50	16	21	28	36	45
Diff=60	12	16	20	26	32
Diff=70	9	12	16	20	24
Diff=80	8	10	13	16	19

NB: Need to add 10% of patients to account for skewness in the distribution of the MRI score

Required sample size for a 95% and with multiplicity adjustment (α = 0.0125) for various value of the target mean differences (Diff) et various assumed value of the standard deviation of differences:

	SD=50	SD=60	SD=70	SD=80	SD=90
Diff=40	31	42	56	72	91
Diff=50	21	28	37	48	59
Diff=60	16	21	27	34	42
Diff=70	13	16	21	26	32
Diff=80	11	13	17	21	25

NB: Need to add 10% of patients to account for skewness in the distribution of the MRI score

⇨ ***We can expect that a total of 48 patients would provide 95% power to detect a difference of 50 units in D-dimer score between start and 12 months with a Wilcoxon's signed-rank test, assuming a standard deviation of differences of 80 and a type I error of 1.25%***

QOL – 3 months versus 12 months
Results observed in the Boscolo et al study are:

- mean difference of 3.33
- standard deviation of differences: 21.6

Required sample size for a 95% and no multiplicity adjustment (α = 0.05) for various value of the target mean differences (Diff) et various assumed value of the standard deviation of differences:

	SD=10	SD=20	SD=25	SD=30	SD=40
Diff=5	54	210	327	470	834
Diff=10	16	54	84	119	210
Diff=15	8	26	39	54	95
Diff=20	6	16	23	32	54
Diff=30	4	8	12	16	26

NB: Need to add 10% of patients to account for skewness in the distribution of the QOL score

Required sample size for a 95% and with multiplicity adjustment (α = 0.0125) for various value of the target mean differences (Diff) et various assumed value of the standard deviation of differences:

	SD=10	SD=20	SD=25	SD=30	SD=40
Diff=5	72	278	433	621	1102

```
Diff=10    21    72   111   158   278
Diff=15    11    34    51    72   126
Diff=20     8    21    31    42    72
Diff=30     6    11    16    21    34
```

NB: Need to add 10% of patients to account for skewness in the distribution of the QOL score

- ***We can expect that a total of 158 patients would provide 95% power to detect a difference of 10 units in QoL score between 3 months and 12 months with a Wilcoxon's signed-rank test, assuming a standard deviation of differences of 30 and a type I error of 1.25%***

of 1.25% (Bonferroni adjustment for the 4 groups). Results are shown for two scenarios each time (one with difference close from the observed one and one with SD close to the observed one).
These results indicate that for VAS, D-Dimer, and MRI:

- ✓ ***We have excellent to sufficient power in the 3 larger groups (A, B, C) for 3 parameters (VAS, D-Dimer, MRI), where the power is clearly higher than the one achieved in the pilot study. Meaning that a smaller difference can be statistically detected if the SD remains the same; or equivalently that a difference of the same order can be detected even of the SD is larger. This is however boarderline in group C for MRI.***
- ✓ ***On the other hand, the power is clearly to low in the three remaining group (D,E,F) and is just reaching 90% when grouping these 3 groups (except for MRI for which it remains too low).***

For QoL, only the analysis in the first group (A) reach sufficient power, as the sample size will be large enough to detect a 10% change, even if the SD is slightly larger than the one observed in the pilot study. Unfortunately, the power will be lower than 90% in the other groups to detect 10% change with a SD close to the one observed in the pilot study.
In general the study will be overpowered when analyzing all patients together, reaching 100% power to detected a difference in VAS score of 4 (SD 2.5), a difference in D-dimer of 4500 (SD 4000), a difference in MRI score of 50 (SD 75), and a difference of 10% (SD 25%) in QoL; all with a type I error of 1.25% (therefore adjusting for the 4 parameters of interest, but not for multiplicity due to the groups).

Group	Expected dist.	Expected n	Power 95% - Alpha 1.25%			
			VAS	D-dimer	MRI	QOL
			4.6<br>(SD 2.3)	4859<br>(SD 3933)	60.6<br>(SD 73.8)	3.33<br>(SD 21.6)
ALL	100%	250				
Group A	64%	160	0.9 (SD 2.5)<br>4 (SD 11.5)	1386 (SD 4000)<br>4500 (SD 13000)	28 (SD 80)<br>55 (SD 160)	8.7 (SD 25)<br>10 (SD 29)
Group B	20%	50	1.6 (SD 2.5)<br>4 (SD 6.4)	2532 (SD 4000)<br>4500 (SD 7110)	50 (SD 80)<br>55 (SD 88)	15.8 (SD 25)<br>10 (SD 18)*
Group C	10%	25	2.3 (SD 2.5)<br>4 (SD 4.3)	3726 (SD 4000)<br>4500 (SD 4830)	75 (SD 80)<br>55 (SD 60)	23.3 (SD 25)
Group D	2%	5	11.3 (SD 2.5)	18051 (SD 4000)	361 (SD 80)	112.5 (SD 25)
Group E	2%	5	11.3 (SD 2.5)	18051 (SD 4000)	361 (SD 80)	112.5 (SD 25)
Group F	2%	5	11.3 (SD 2.5)	18051 (SD 4000)	361 (SD 80)	112.5 (SD 25)
D+E+F		15	3.3 (SD 2.5)<br>3.0 (SD 2.5)*	5320 (SD 4000)<br>4845 (SD 4000)*	106 (SD 80)<br>97 (SD 80)*	33 (SD 25)<br>30 (SD 25)*

With (A) Venous malformation, unifocal + multifocal + BRBN + Capillaro venous malformation ; (B) LM + LVM ; (C) CLVM + KTS + CLOVES ; (D) Gorham + GLA; (E) PHTS; (F) KHE + tufted angioma
Accounting 10% of patients to take into account non-normality (skewness) of the data
* Power of 90%

NB: When interpreting these results, one have to keep in mind that

- ⇨ A higher SD then the one assume in the sample size calculation will lead to a lower power
- ⇨ A smaller true difference then the one assume in the sample size calculation will lead to a lower power

Note: All sample size calculation has been performed using the “power.t.test” function of the R software.

B) Supplemental methods: Detailed genetic analysis

DNA was quantified using NanoDrop 8000 (Thermo Fisher Scientific) and Qubit 2.0 (Thermo Fisher Scientific). We used two different amplification-based panels: an Ion AmpliSeq panel for targeted sequencing of all the coding exons of the *TIE2* and *PIK3CA* genes including ten nucleotides of all flanking introns (www.ampliseq.com) and a QIASeq Targeted DNA Custom Panel for the analysis of the exons 17, 22, 23 of *TIE2* and all the coding exons of *PIK3CA* including twenty-five nucleotides of all flanking introns (Qiagen; www.qiagen.com). The analysis was done using one or the other of the two panels for each sample. The Ion AmpliSeq panel consisted of 2 pools of primers for multiplexed PCR-amplification with Ion Ampliseq Library kit, and sequencing on an Ion Personal Genome Machine (PGM) or an ion Proton (Thermo Fisher Scientific). Reads were aligned to the human reference sequence hg19, using the Torrent Suite Server. Bam files were imported into Highlander software package (https://sites.uclouvain.be/highlander/) for analysis. For the QIASeq custom panel, the library preparation was done using the Qiagen Library kit and the sequencing was performed on a MiSeq instrument (Illumina, San Diego, CA, USA). The genetic raw data (FastQ files) were imported into the SOPHIA DDM (Saint-Sulpice, Switzerland) software platform version 5.10 for bioinformatics analysis (alignment and variant calling). The variant calling provided by the SOPHIA GENETICS platform was specifically optimized for the use of the custom “QIASeq Targeted DNA Custom Panel” (Qiagen). We selected variants with at least 5 mutant reads representing at minimum

1% of all alleles by interrogating all positions reported with at least 4 changes in the COSMIC database (https://cancer.sanger.ac.uk/cosmic). Samples needed to have an average coverage above 500x to be considered not to contain a mutation.

C) Supplementary Table

Table 1S

Detailed baseline characteristics

**Patient characteristics**	n = 132	%
Male	46	35
Female	86	65
Median age (range)	30 (0-73)	
Age at inclusion		
- 0-12	23	17
- 13-18	8	6
- 19-40	56	42
- 41-60	36	27
- >60	9	7
**Venous malformations**	**76**	**58**
- Sporadic Venous Malformation (VM)	74	56
- Blue Rubber Bleb Naevus syndrome (BRBN)	1	1
- Glomuvenous malformation	1	1
**Capillary malformations**	**7**	**5**
- Diffuse capillary malformation with overgrowth (DCMO)	3	2
- Capillary malformation with dilated veins (CMDV)	4	3
**Lymphatic malformation (LM)**	**27**	**20**
- Lymphatic malformation (LM)	21	16
- Generalized Lymphatic Anomaly (GLA)	6	5
**Combined vascular malformation**	**22**	**17**
- Lymphatico-Venous Malformation (LVM)	1	1
- Capillaro-Venous Malformation (CVM)	5	4
- Klippel-Trenaunay Syndrome (KTS)	5	4
- Capillaro-Lymphatico-Venous Malformation (CLVM)	2	2
- CLOVES syndromes	4	3
- PTEN Hamartoma Tumor Syndrome (PHTS)	3	2
- Gorham-Stout Disease (GSD)	2	1
**Anatomical Region**		
Cervico-facial	38	29
Trunk	6	5
Limb	56	42
Cervicofacial and trunk	2	2
Trunk and limb	21	16
Cervicofacial, trunk and limb	6	5
Internal localization	3	2
**Extension**		
Localized / Extensive	70 / 62	53 / 47
Superficial / Deep	9 / 123	7 / 93
**Continuous Pain**		
- No pain	31	23
- Low (1-3)	14	11

- Moderate (4-6)	53	40
- High (7-10)	34	26
**Pain Exacerbation**		
- No pain exacerbation	28	21
- 1-2 crisis/month	63	48
- 3-4 crisis/month	25	19
- 5 or more crisis/month	16	12
**Intensity of exacerbation**		
- No pain exacerbation	28	21
- Low (1-3)	4	3
- Moderate (4-6)	23	17
- High (7-10)	77	58
**Functional Limitation**		
- No functional limitation	26	20
- Low (1-3)	17	13
- Moderate (4-6)	47	36
- Strong (7-10)	42	32
**Other symptoms**		
- None	97	73
- Bleeding	8	6
- Infection	6	5
- Oozing	4	3
- Ulceration	4	3
- Multiple symptoms	13	10
**Deformation**		
- No	54	41
- Yes	78	59
**Prior radical treatment**		
None	44	33
Surgery	24	18
Sclerotherapy	27	20
Surgery + sclerotherapy	37	28
**Interval between last radical treatment and inclusion (n=79)**		
<2 year	27	31
2-5 years	24	27
>5 years	37	42
**Concurrent medical treatment**		
- Elastic garment	32	24
- HBPM	24	18
- Painkiller	93	70
- Level 1 (Paracetamol / AINS)	91	69
- Level 2 (Codeine, tramadol)	34	26
- Level 3 (Narcotic analgesics)	0	0

Table 2S

Genetic analysis of vascular malformations

	VM	CVM	DCMO	CMDV	CLVM	BRBN	CLOVES	KTS	LM	PHTS	GVM	**Total**
TIE2	18					1						**19**
PIK3CA	9	2		1	2		3	3	4			**24**
PTEN										3		**3**
GNAQ		1	1	2								**4**
GLMN											1	**1**
GNA11			1									**1**

D) Supplementary Figure 1S

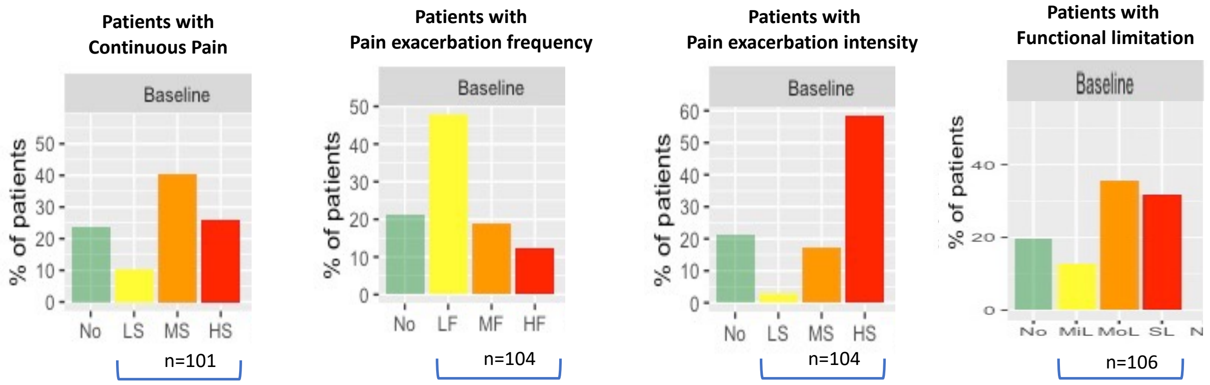

Figure 1S: Patient population clinical symptoms at baseline: Pain score **(A)**, pain exacerbation frequency **(B)** and pain exacerbation intensity scores **(C)** measured at baseline. Pain score and pain exacerbation intensity score presented as no pain (No), low score (LS) from 1 to 3, medium score (MS) from 4 to 6 and high score (HS) from 7 to 10. Pain exacerbation frequency presented as no pain exacerbation (No), low frequency of 1 to 2 crises per month (LF), mild frequency of 3 to 4 crises per month (MF) and high frequency of 5 or more crises per month (HF). Patients with missing information or out of study were grouped under No available information (NA). Functional limitation **(D)** measured at baseline expressed as no functional limitation (No), mild limitation score of 1 to 3 (MiL), moderate limitation score of 4 to 6 (MoL) and strong limitation score of 7 to 10 (SL).

E) Study protocol (Initial version 2015)

Phase III multicentric study evaluating the efficacy and safety of sirolimus in Vascular Anomalies that are refractory to standard care

Sponsor: Cliniques Universitaires Saint-Luc, Université Catholique de Louvain, avenue Hippocrate 10, 1200 Brussels

Protocol identification number: VASE

EudraCT number: 2015-001703-32

ClinicalTrials.gov Identifier:

MedDRA classification code: Diseases [C] – D18

Version 1.1 in date of 24.09.2015

CONTACT:

Multicenter study

Coordinator site

Center for Vascular Anomalies
Division of Plastic Surgery
Cliniques Universitaires Saint-Luc,
Université Catholique de Louvain,
Brussels, Belgium

Study coordinator
Principal investigators

Laurence M. Boon, MD, PhD
Center for Vascular Anomalies
Division of Plastic Surgery
Cliniques Universitaires Saint-Luc,
Université Catholique de Louvain,
10, Avenue Hippocrate
1200 Brussels
Tel: + 32-2-764 1403
Fax: + 32-2-764 7460
E-mail: laurence.boon@uclouvain.be

Emmanuel Seront, MD, PhD (co-PI)
Institut Roi Albert II

Medical Oncology
Cliniques Universitaires Saint-Luc,
Université Catholique de Louvain,
10, Avenue Hippocrate
1200 Brussels
Tel: + 32-2-764 5102
E-mail: emmanuel.seront@uclouvain.be

Co-Investigators:

Professor Prof. Dr. Jochen Rößler
Leitender Oberarzt (Funk 12-4514)
Pädiatrische Hämatologie und Onkologie
Zentrum für Kinder- und Jugendmedizin
UNIVERSITÄTSKLINIKUM FREIBURG
Mathildenstr. 1, 79106 Freiburg
Tel: +49 761 270-43000 (Information)
Fax: +49 761 270-45180
Email: jochen.roessler@uniklinik-freiburg.de

Dr Anne Dompmartin-Blanchère, MD, PhD
Pôle Médecine d'Organes et Cancérologie Médicale Service de Dermatologie CHU Clemenceau 14033 CAEN Cedex 9 02 31 27 25 10
Tel: +33 6 62 32 30 57
Email: dompmartin-a@chu-caen.fr

Dr Samira Batul Syed
MBBS DCH DCCH RCPEd RCGP FCM BTEC Adv LASER DPD (2012)
Lead for the Staff and Associate Specialists Group at GOSH Associate Specialist in Paediatrics.(Dermatology)
Skin laser/Birthmark/Vascular Anomalies Unit.
Tel: 0207 405 9200 Extension 0064 Bleep 0909
Email: SamiraBatul.Syed@gosh.nhs.uk

Dr Eulalia Baselga
Cap Clínic, Unitat de Dermatologia Pediàtrica
Professora Associada
Departament de Dermatologia
Hospital de la Santa Creu i Sant Pau.
Universitat Autònoma de Barcelona
Mas Casanovas 90
08041 Barcelona,
Tel: +34 935537007
Fax: +34 935537008
Email: dra.baselga@gmail.com

Dr Maya El Hachem
Director, Dermatology Unit
Coordinator of the vascular anomalies activities

Children's Hospital Bambino Gesù, IRCCS
Piazza San Onofrio, 4
00165, Rome- Italy
President of the Italian Society for the Study of Vascular Anomalies (SISAV)
Tel: 066859-2020/2509/2597
Fax: 0668592300
Email: may.elhachem@opbg.net

Dr Päivi Salminen,MD
Division of Paediatric Surgery
Children´s Hospital Helsinki
Cordinator of Vascular Anomaly Team
Helsinki University Hospital
Stenbäckinkatu 11
00290 Helsinki, Finland
Email: Paivi.Salminen@hus.fi

Dr Annouk Bisdorff Bresson
Hôpital Lariboisiere
Service de Neuroradiologie de Pr Houdart
Consultation des Angiomes
2, rue Ambroise Pare
75010 Paris, France
Tel: 0033-1 49 95 95 84 50
Email: annouk.bisdorff@aphp.fr

Prof Isabelle Quere
Service de Médecine Interne et Maladies Vasculaires
EA2992 - Université Montpellier I
CHU Saint Eloi
80 Avenue Auguste Fliche
304295 Montpellier Cedex 5, France
Tel: 0033-467 33 70 25
Fax: 0033-467 33 70 23
Email: i-quere@chu-montpellier.fr

Prof Alan Irvine
Pediatric Dermatology
Our Lady's Children's Hospital
Crumlin
Dublin 12, Ireland
Tel: 00353 1 416 21 03
Email address: irvinea@tcd.ie

LIST OF ABBREVIATIONS:

AE Adverse Event
AFMPS Agence Fédérale des Médicaments et des Produits de Santé
Akt See PKB (Protein Kinase B)
ALT/GPT ALanine aminoTransferase/Glutamic Pyruvic Transaminase
ANC Absolute Neutrophil Count
AST/GOT ASpartate aminoTransferase/Glutamic Oxaloacetic Transaminase
AUC Area Under the concentration-time Curve
$AUC_{t,ss}$ Area Under the concentration-time Curve from Time zero to Steady-State
AVF ArterioVenous Fistula
AVM ArterioVenous Malformation
BMI Body Mass Index
C_{max} Maximum serum Concentration
$C_{max,ss}$ Maximum (peak) Steady-State plasma drug Concentration during a dosage interval
$C_{min,ss}$ Minimum Steady-State plasma drug Concentration during a dosage interval
CM-AVM Capillary Malformation -ArterioVenous Malformation
CEBHF Comité d'Ethique Biomédicale Hospitalo-Facultaire
CL/F Clearance
CLM Capillary-Lymphatic Malformation
CLVM Capillary-Lymphatico-Venous Malformation
CM Capillary Malformation
CPK Creatine PhosphoKinase
CR Complete Response
CRF Case Report/Record Form
CT Computed Tomography
CTCAE Common Terminology Criteria for Adverse Events
CVM Capillary-Venous Malformation
CYP Cytochrome P450
DLCO Diffusing capacity or transfer factor of the Lung for Carbon Monoxide
DLT Dose Limiting Toxicity
ECG ElectroCardioGram
EGFR Epithelial Growth Factor Receptor
EV EudraVigilance (code)
FDA Food and Drug Administration
FK FK506 (calcineurin activation blocker)
FKBP(-12) FK-Binding Protein 12
FPG Fasting Plasma Glucose
GCP Good Clinical Practice
GLA Generalized Lymphatic Anomalies
HDL High-Density Lipoprotein
Hgb Hemoglobin
HHT Hereditary Hemorrhagic Telangiectasia
HIV Human Immunodeficiency Virus
HMG-Coa 3-Hydroxy-3-MéthylGlutaryl-Coenzyme A
HPF High-Powered Field
ICH International Conference on Harmonization
IEC Independent Ethics Committee

INR International Normalized Ratio
IRB Institutional Review Board
ISSVA International Society for the Study of Vascular Anomalies
KHE Kaposiform HemangioEndotheliomas
KMP Kasabach–Merritt Phenomenon
LC-MS/MS Liquid Chromatography and Tandem Mass Spectrometry
LDH Lactate Dehydrogenase
LDL Low-Density Lipoprotein
LFT Liver Function Test
LIC Localized Intravascular Coagulopathy
LLN Lower Limit of Normal
LM Lymphatic Malformation
LSLV Last Patient Last Visit
LVEF Left Ventricular Ejection Fraction
LVM Lymphatico-Venous Malformation
MedDRA Medical Dictionary for Regulatory Activities
MOS SF-36 Medical Outcome Study Short Form-36
MRI Magnetic Resonance Imaging
mTOR Mammalian Target Of Rapamycin
mTORC Mammalian Target Of Rapamycin Complex
P-4EBP1 Phospho-eukaryotic translation initiation factor 4E-Binding Protein 1
P-gp P-glycoprotein
PI Principal Investigator
PI3K PhosphatidylInositol-3-Kinase
PKB Protein Kinase B (or Akt)
PLT Platelets
PTEN Phosphatase and Tensin Homolog
QTc QT interval (corrected)
QTcF QT interval corrected for heart rate using Fridericia's formula
RAP Reporting and Analysis Preparation
S6 Ribosomal Protein S6
S6K S6 Protein Kinase
SAE Serious Adverse Event
SD Standard Deviation
t1/2 Half-life
t_{max} Time to reach maximum (peak) plasma concentration
$t_{max,ss}$ Time to reach maximum (peak) plasma concentration following drug administration at Steady-State
TSC1/2 Tuberous Sclerosis Complex
TSH Thyroid Stimulating Hormone
ULN Upper Limit of Normal
VAS Visual Analog Scale
VM Venous Malformation
WBC White Blood Count
WHO World Health Organization

ABSTRACT:

Vascular malformations are rare structural abnormalities of blood and lymphatic vessels that occur during vasculogenesis, lymphovasculogenesis, angiogenesis and lymphangiogenesis. Based on clinical, radiological and biological criteria, they are divided depending on the affected vessels into arteriovenous, capillary, lymphatic, venous or combined malformations. They can affect any tissues and any organs. Depending on their size and location, they can cause deformation, severe pain, chronic anemia and severe functional restraint. As conventional treatments (medical, surgical resection and/or sclerotherapy) are seldomly curative, there is a need for new therapeutic tools.

Sirolimus is an immunosuppressive drug used in organ transplantations. It inhibits mTOR protein, which is part of the PI3K/AKT/mTOR pathway, downstream of various tyrosine kinase receptors, including VEGFRs and TIE2. Therefore sirolimus can intervene in the growth and organization of the vascular and lymphatic system. One study on a limited number of patients (n=6) has been published and it demonstrated an important improvement in the quality of life of these patients with debilitating vascular malformations [18]. In a pilot study including few selected patients with lymphatic malformation, venous malformation or complex vascular malformations that were refractory to standard care, we detected that sirolimus can reduce pain, functional impairment, bleeding, intravascular coagulopathy and lesion size in most of them [9][Hammer et al, in preparation]. Because of the limited number of patients in this pilot study, a larger study is now warranted in a wider range of clinical phenotypes and more important number of patients, in order to be able to perform statistical analysis for various signs and symptoms, evaluate long-term outcome and give an algorithm for the management of these complicated vascular malformations.

The primary objectives of our prospective multicentric study are 1) to assess the potential statistical efficacy on signs and symptoms caused by these complex vascular anomalies that are refractory to standard care, 2) to evaluate the effect on the quality of life of the patient and 3) to see whether this treatment can statistically reduce the coagulation abnormalities observed in patients with venous anomalies. The secondary objectives are 1) to confirm the security of this treatment, 2) to see which patients would benefit the most, and thus indicated for sirolimus in the future and 3) to see whether this treatment will reduce the volume of the malformation (on MRI) on a long-term follow-up.

All patients will be evaluated on a monthly basis for the first 3 months and then every 3 months by the PI for the evaluation of the effects of the medication on the malformation (reduction of signs and symptoms, modification of the quality of life) and by an adult (co-PI) or pediatric oncologist for the administration and safety of the medication. Blood test will be done to titrate the medication, to exclude any side effects, to evaluate the initial anemia and the coagulation abnormality associated with some vascular malformations. An MRI will be performed on a yearly basis to evaluate the evolution of the volume of the malformation under sirolimus treatment. A total of 250 patients will be included for a duration of 2 years of medication, and a subsequent follow-up of 5 years.

TABLE OF CONTENTS:

1. INTRODUCTION
   1.1 Background
      1.1.1 Overview of vascular malformations and treatment
      1.1.2 Vascular malformations and PI3K/Akt/mTOR pathway
      1.1.3 Study rationality
   1.2 Clinical experience and safety of sirolimus
      1.2.1 Pharmacodynamics
      1.2.2 Pharmacokinetics
      1.2.3 Metabolism
      1.2.4 Clinical efficacy
      1.2.5 Clinical safety and tolerability
2. TRIAL JUSTIFICATION
3. TRIAL OBJECTIVES
   3.1 Purpose
   3.2 Primary objectives
   3.3 Secondary objectives
4. TRIAL DESIGN
   4.1 Screening phase
   4.2 Enrollment
   4.3 Treatment phase/duration of treatment
   4.4 End of treatment
   4.5 Follow-up phase
   4.6 Additional tests
5. POPULATION-INCLUSION AND EXCLUSION CRITERIA
6. STUDY DRUG
   6.1 Allocation to treatment
   6.2 Study drug
      6.2.1 Dosing regimen
      6.2.2 Preparation of the study drug
      6.2.3 Special considerations
      6.2.4 Known undesirable effects of sirolimus
   6.3 Dose modifications and interruption and treating adverse events
      6.3.1 Guidelines for continuation of treatment
      6.3.2 Management of stomatitis/oral mucositis/mouth ulcers
      6.3.3 Management of hyperlipidemia and hyperglycemia
      6.3.4 Management of diarrhea
      6.3.5 Headache
      6.3.6 Management of non-infectious pneumonitis
   6.4 Treating the patient and dose modifications
      6.4.1 Sirolimus dosing instructions
      6.4.2 Dose modifications
      6.4.3 Follow-up for toxicities
   6.5 Concomitant Medication(s)
   6.6 Study treatment discontinuation
   6.7 Premature patient withdrawal
7. VISIT SCHEDULES
8. EVALUATION CRITERIA-EFFICACY AND SAFETY
   8.1 Response assessment

8.2 Side effects assessment
9. ADVERSE EVENT REPORTING
9.1 Adverse events
9.2 Reporting period
9.3 Definition of an Adverse Event
9.4 Abnormal test findings
9.5 Serious Adverse Events
9.6 Hospitalization
9.7 Severity assessment
9.8 Causality assessment
9.9 Adverse event Reporting Requirements
9.10 Serious Adverse Event Reporting Requirements
10. QUALITY CONTROL AND QUALITY ASSURANCE
11. DATA HANDLING AND RECORD KEEPING
11.1 Case Report Forms
11.2 Record retention
12. ETHICS
12.1 Institutional Review Board (IRB)/Independent Ethics Committee (IEC)
12.2 Ethical conduct of the trial
12.3 Subject information and consent
13. INSURANCE AND FUNDING
13.1 Insurance
13.2 Funding
14. DEFINITION OF END OF TRIAL
15. PUBLICATION OF STUDY RESULTS
16. REFERENCES
17. APPENDICES

1. INTRODUCTION:

1.1 Background

1.1.1 Overview of vascular malformations and treatments

Vascular Malformations are rare structural abnormalities that develop during vasculogenesis and/or lymphangiogenesis. Based on clinical, radiological, histopathological and haemodynamic characteristics, they are divided into fast-flow and slow-flow malformations and, depending on the affected malformed vessels, into arteriovenous (AVM), capillary (CM), lymphatic (LM) and venous malformations (VM) [1-4]. Any combination is possible, causing complex/combined vascular malformations; many of which being known as eponymous syndromes [5-6]. Depending on their size and location, vascular malformations can induce disfigurement, acute and chronic pain, as well as organic dysfunction causing significant morbidity and mortality.

Venous malformations (and combined lesions with a venous component) are the most common referral to specialized Center for Vascular Anomalies. They can affect any tissue (skin, sub-cutaneous tissue, muscle, nerve, bone) and any organ (e.g.gastrointestinal, pulmonary, cerebral and genital). They often cause pain and functional limitation and sometimes bleeding. They predispose to venous stasis and localized intravascular coagulopathy (LIC) [7]. Chronic LIC can result in phlebolith formation, pain and per and postoperative bleeding due to decompensation into disseminated intravascular coagulopathy. Mutations in the *TIE2* gene have been identified as the cause of 50% of sporadic venous malformations [8]. Moreover studies on mouse model recapitulating human VM showed that sirolimus can prevent lesion enlargement.

Lymphatic malformations can be macro or microcystic. They can be well-localized or diffuse, like in Generalized Lymphatic Anomalies (GLA). They often lead to significant disfigurement from soft tissue hypertrophy to skeletal overgrowth. They are commonly responsible for oozing, bleeding and recurrent cellulitis that can lead to septicemia. They can also cause bony abnormalities and destruction, chylous ascites and pericardial or pleural effusions [9]. Mutations in the *PIK3CA* gene encoding the kinase domain of Phosphoinositol 3 Kinase (PI3K) have been identified [10].

Combined slow-flow malformations are more rare. Depending on the affected vessel and location, it can cause deformation, pain, chronic anemia, functional impairment and sometimes threaten life. Klippel-Trenaunay syndrome (KTS) is the most known eponym that is characterized by capillary-lymphatico-venous malformation with overgrowth of the extremity. Patient with KTS are at high risk of pulmonary embolism. Proteus syndrome, also known as PTEN-hamartoma lesion is another rare syndrome that can present with slow-flow or high-flow vascular malformation. These patients are at high risk of developing malignant cancer. Mutations in PTEN (Phosphatase and Tensin homolog) gene have been identified in this syndrome [11].

<u>Vascular malformations: treatment</u>

Management of these malformations is complex and necessitates a multidisciplinary specialized center as it includes various modalities such as medical, radiological and surgical treatments. Medical treatment includes pain medication, compression garments, low molecular weight heparin, aspirin and aminocaproic acid. Some lymphatic malformations have been

treated, with poor or no efficacy, with interferon alone [12] or in combination with bisphosphonates for bony disease [9], or other agents such as cyclophosphamide [13,14]. Sclerotherapy is the gold standard management for venous malformations and for some lymphatic malformations. It needs to be performed by an experienced interventional radiologist as sclerosing agents are not specific. Major complications can occur such as ulceration, nerve paralysis and even death. [15]. Surgical resection can be done in well-localized venous and/or lymphatic malformations. In extensive malformation, recurrence is common as complete resection will cause major functional and esthetic impairment.

Nevertheless, many patients affected with extensive venous, lymphatic and/or combined malformations are still in tremendous pain and present severe functional limitation despite severe dose of pain medication, optimal medical treatment and several interventional and/or surgical treatment sessions. Therefore, it is evident that better treatments are mandatory in order to help these patients that are debilitated by these extensive slow-flow malformations.

1.1.2 Vascular malformations and PI3K/Akt/mTOR pathway

Recent preclinical studies have shown the important role of the PI3K/AKT (also known as PKB, Protein Kinase B)/mammalian target of rapamycin (mTOR) pathway (**Figure 1**) on the development and the lymphatic-vascular organization, suggesting an appealing therapeutic target to treat patients with complex vascular malformations.

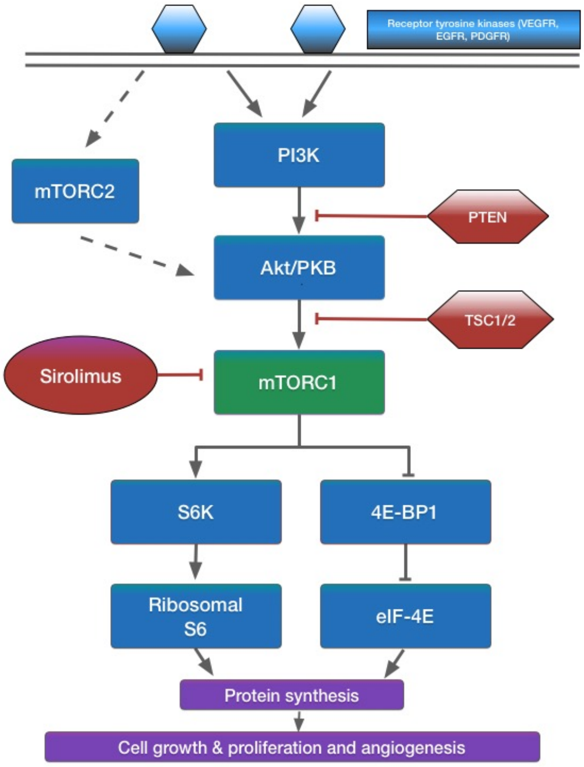

Fig. 1. PI3K/Akt/mTOR pathway

The mTOR protein is a serine/threonine kinase which is composed of two complexes, mTOR complex 1 (mTORC1) and mTOR complex 2 (mTORC2). This pathway plays an important

role in cell growth, proliferation, survival and angiogenesis through sensing and integrating energetic signals from cellular environment.
In response to nutrients or growth factors (such as Vascular Endothelial Growth Factor (VEGF), Epidermal Growth factor (EGF), Platelet derived Growth factor (PDGF)) binding to their specific receptor (VEGF receptor, EGF receptor, PDGF receptor), mTORC1 is activated through the PI3K/Akt cascade, leading to phosphorylation of S6-kinase 1 (S6K1) and p-4EBP1 (eukaryotic translation initiation factor 4E (eIF4E)-binding protein), two proteins implicated in activation of translation enzymatic machinery. This mTOR activation stimulates also the angiogenesis process via HIF and VEGF production. The role of mTORC2 is less well known but it is well demonstrated that mTORC2 could stimulate directly Akt. Akt can influence cellular proliferation independently of mTOR activation, inactivating cell cycle inhibitors, promoting progression through the G1-S cell cycle check point, and mediating inhibition of pro-apoptotic genes and degradation of the tumor suppressor protein p53.

Dysregulation of any steps along the PI3K/Akt/mTOR pathway can result in excessive and unrepressed activation of mTOR leading to tumor development and uncontrolled angiogenesis. The PI3K/Akt/mTOR pathway is controlled principally by PTEN, a phosphatase protein encoded by PTEN gene, which prevents the Akt activation by PI3K [16]. The tumor suppressor proteins TSC1 and 2 exert also negative effects upon this pathway, acting as a brake of mTOR activation.

Other members of the PI3K/mTOR pathway have also been implicated in the generation and propagation of vascular anomalies. Vascular endothelial growth factor (VEGF) is a key regulator in lymphangiogenesis and angiogenesis, and acts as both a potential upstream stimulator and a downstream effector of mTORC1. Akt has been found to be over-expressed in the endothelial cells of cutaneous vascular malformations in a murine model [17]. Mutations in *PTEN* have been identified in both fast-flow vascular anomalies and in slow-flow lesions with associated overgrowth [11].

Sirolimus, also known as rapamycin, is a direct allosteric inhibitor of mTORC1, thereby preventing the binding of mTORC1 to its targets (S6K1 and 4E-BP1), resulting in inhibition of protein synthesis, cell proliferation and angiogenesis. The mode of action of sirolimus is to bind the cytosolic protein FK-binding protein 12 (FKBP12) in a manner similar to tacrolimus. Unlike the tacrolimus-FKBP12 complex, which inhibits calcineurin, the sirolimus-FKBP12 complex inhibits the mTOR pathway by directly binding to mTORC1. Sirolimus must bind FKBP12 first, and only the FKBP12-sirolimus complex can bind mTOR.

1.1.3 Study rationality

mTOR inhibitors, such as sirolimus, have been postulated to be beneficial in the treatment of complex vascular malformations that are resistant to conventional management. Previous study on a limited number of patients (n=6) affected with lymphatic malformations have already been published and demonstrate an important improvement in the quality of life of these patients with debilitating vascular malformations [18]. Treatment of Proteus syndrome (resulting from a PTEN mutation) by oral sirolimus has been the object of a case report [19]. These patients had multiple hamartomas leading to respiratory and gastrointestinal dysfunction that showed clinical improvement within 2 months of sirolimus medication. Blue Rubber Bleb Naevus syndrome, characterized by multiple cutaneous venous malformations associated with gastrointestinal location and chronic anemia has also shown clinical improvement with sirolimus [20].

Our initial pilot study including a few selected patients with lymphatic malformations, venous malformations or complex vascular malformations that were refractory to standard care, detected that sirolimus can reduced pain, functional impairment, bleeding, intravascular coagulopathy and lesion size in most of them [9] (Hammer al, in preparation).

1.2 Clinical experience and safety of sirolimus

Sirolimus is currently the only FDA-approved mTOR inhibitor. It is indicated for prevention of kidney allograft rejection in adults and children above 13 year of age, but is commonly used to manage organ rejection in younger children. In renal transplantation, sirolimus has been well tolerated at a through level of 15–20 ng/ml, with some hyperlipidemia [21]. In one study of liver and small bowel transplants, 77% of patients tolerated the drug without incident [22]. In a pediatric renal transplant study, forty-nine children were treated with sirolimus, with the main toxicities being hyperlipidemia, mucositis, and poor wound healing [23]. Sirolimus derivatives, such as everolimus and temsirolimus, are also approved in different kinds of cancer including renal, neuroendocrine and breast cancers [24-27].

1.2.1 Pharmacodynamics

Pharmacotherapeutic group: Immunosuppressants, selective immunosuppressants.
Sirolimus inhibits T-cell activation and proliferation of other cells induced by most stimuli, by blocking calcium-dependent and calcium-independent intracellular signal transduction. Studies demonstrated that its effects are mediated by a mechanism that is different from that of ciclosporin, tacrolimus, and other immunosuppressive agents. Experimental evidence suggests that sirolimus binds to the specific cytosolic protein FKBP-12, and that the FKBP 12-sirolimus complex inhibits the activation of mTOR. The inhibition of mTOR results in blockage of several specific signal transduction pathways, thereby preventing downstream protein synthesis and subsequent cell proliferation and angiogenesis. The inhibition of lymphocyte activation results in immunosuppression.

1.2.2 Pharmacokinetics (based on the data of the European Medicines Agency EMA)

Oral solution

Following administration of the sirolimus oral solution, sirolimus is rapidly absorbed, with a time to peak concentration of 1 hour in healthy subjects receiving single doses and 2 hours in patients with stable renal allografts receiving multiple doses. The systemic availability of sirolimus in combination with simultaneously administered ciclosporin (Sandimune) is approximately 14%. Upon repeated administration, the average blood concentration of sirolimus is increased approximately 3-fold. The terminal half-life in stable renal transplant patients after multiple oral doses was 62 ± 16 hours. The effective half-life, however, is shorter and mean steady-state concentrations were achieved after 5 to 7 days.

After a single dose of [$_{14}$C] sirolimus in healthy volunteers, the majority (91.1%) of radioactivity was recovered from the faeces, and only a minor amount (2.2%) was excreted in urine. Clinical studies of sirolimus did not include a sufficient number of patients above 65 years of age to determine whether they will respond differently than younger patients. Sirolimus trough concentration data in 35 renal transplant patients above 65 years of age were similar to those in the adult population (n=822) from 18 to 65 years of age.

In pediatric patients on dialysis (30% to 50% reduction in glomerular filtration rate) within age ranges of 5 to 11 years and 12 to 18 years, the mean weight-normalized CL/F was larger for younger pediatric patients (580 ml/h/kg) than for older pediatric patients (450 ml/h/kg) as compared with adults (287 ml/h/kg). There was a large variability for individuals within the age groups.

Sirolimus concentrations were measured in concentration-controlled studies of pediatric renal-transplant patients who were also receiving ciclosporin and corticosteroids. The target for trough concentrations was 10-20 ng/ml. At steady-state, 8 children aged 6-11 years received mean ± SD doses of 1.75 ± 0.71 mg/day (0.064 ± 0.018 mg/kg, 1.65 ± 0.43 mg/m_2) while 14 adolescents aged 12-18 years received mean ± SD doses of 2.79 ± 1.25 mg/day (0.053 ± 0.0150 mg/kg, 1.86 ± 0.61 mg/m_2). The younger children had a higher weight-normalized CL/F (214ml/h/kg) compared with the adolescents (136 ml/h/kg). These data indicate that younger children might require higher bodyweight-adjusted doses than adolescents and adults to achieve similar target concentrations. However, the development of such special dosing recommendations for children requires more data to be definitely confirmed.

In mild and moderate liver impaired patients (Child-Pugh classification A or B), mean values for sirolimus AUC and $t_{1/2}$ were increased 61% and 43%, respectively, and CL/F was decreased 33% compared to normal healthy subjects. In patients with severe liver impaired patients (Child-Pugh classification C), mean values for sirolimus AUC and $t_{1/2}$ were increased 210% and 170%, respectively, and CL/F was decreased by 67% compared to normal healthy subjects. The longer half-lives observed in liver impaired patients delay reaching steady-state. The pharmacokinetics of sirolimus were similar in various populations, with renal function ranging from normal to absent (dialysis patients).

<u>Oral tablet</u>

The 0.5 mg tablet is not fully bioequivalent to the 1 mg, 2 mg and 5 mg tablets when comparing C_{max}. Multiples of the 0.5 mg tablets should therefore not be used as a substitute for other tablet strengths.

In healthy subjects, the mean extent of bioavailability of sirolimus after single-dose administration of the tablet formulation is about 27% higher relative to the oral solution. The mean C_{max} was decreased by 35%, and mean t_{max} increased by 82%. The difference in bioavailability was less marked upon steady-state administration to renal transplant recipients, and therapeutic equivalence has been demonstrated in a randomized study of 477 patients. When switching patients between oral solution and tablet formulations, it is recommended to give the same dose and to verify the sirolimus through concentration 1 to 2 weeks later to assure that it remains within recommended target ranges. Also, when switching between different tablet strengths, verification of trough concentrations is recommended.

In 24 healthy volunteers receiving sirolimus tablets with a high-fat meal, C_{max}, t_{max} and AUC showed increases of 65%, 32%, and 23%, respectively. To minimize variability, sirolimus tablets should be taken consistently without food. Grapefruit juice affects CYP3A4-mediated metabolism and must, therefore, be avoided.

Clinical studies of sirolimus did not include a sufficient number of patients above 65 years of

age to determine whether they will respond differently than younger patients. Sirolimus tablets administered to 12 renal transplant patients above 65 years of age gave similar results to adult patients (n=167) 18 to 65 years of age.

Initial therapy (2 to 3 months post-transplant): In most patients receiving sirolimus tablets with a loading dose of 6 mg followed by an initial maintenance dose of 2 mg, whole blood sirolimus trough concentrations rapidly achieved steady-state concentrations within the recommended target range (4 to 12 ng/ml, chromatographic assay). Sirolimus pharmacokinetic parameters following daily doses of 2 mg sirolimus tablets administered in combination with ciclosporin microemulsion (4 hours prior to sirolimus tablets) and corticosteroids in 13 renal transplant patients, based on data collected at months 1 and 3 after transplantation, were: $C_{min,ss}$ 7.39 ± 2.18 ng/ml; $C_{max,ss}$ 15.0 ± 4.9 ng/ml; $t_{max,ss}$ 3.46 ± 2.40 hours; $AUC_{t,ss}$ 230 ± 67 ng.h/ml; CL/F/WT, 139 ± 63 ml/h/kg (parameters calculated from LC-MS/MS assay results). The corresponding results for the oral solution in the same clinical study were $C_{min,ss}$ 5.40 ± 2.50 ng/ml, $C_{max,ss}$ 14.4 ± 5.3 ng/ml, $t_{max,ss}$ 2.12 ± 0.84 hours, $AUC_{t,ss}$ 194 ± 78 ng.h/ml, CL/F/W 173 ± 50 ml/h/kg. Whole blood trough sirolimus concentrations, as measured by LC/MS/MS, were significantly correlated (r_2=0.85) with $AUC_{t,ss}$.

Based on monitoring in all patients during the period of concomitant therapy with ciclosporin, mean (10_{th}, 90_{th} percentiles) troughs (expressed as chromatographic assay values) and daily doses were 8.6 ± 3.0 ng/ml (5.0 to 13 ng/ml) and 2.1 ± 0.70 mg (1.5 to 2.7 mg), respectively (see section 4.2).

Maintenance therapy: From month 3 to month 12, following discontinuation of ciclosporin, mean (10_{th}, 90_{th} percentiles) troughs (expressed as chromatographic assay values) and daily doses were 19 ± 4.1 ng/ml (14 to 24 ng/ml) and 8.2 ± 4.2 mg (3.6 to 13.6 mg), respectively (see section 4.2). Therefore, the sirolimus dose was approximately 4-fold higher to account for both the absence of the pharmacokinetic interaction with ciclosporin (2-fold increase) and the augmented immunosuppressive requirement in the absence of ciclosporin (2-fold increase).

1.2.3 Metabolism

Sirolimus is extensively metabolized by the cytochrome P450 3A4 (CYP3A4) isoenzyme in the intestinal wall and liver. Sirolimus is also a substrate for the multidrug efflux pump, P-glycoprotein (P-gp) located in the small intestine. Therefore, substances that affect these proteins may influence absorption and the subsequent elimination of sirolimus. Inhibitors of CYP3A4 (such as ketoconazole, voriconazole, itraconazole, telithromycin, or clarithromycin) decrease the metabolism of sirolimus and increase sirolimus levels. Inducers of CYP3A4 (such as rifampin or rifabutin) increase the metabolism of sirolimus and decrease sirolimus levels. Co-administration of sirolimus with strong inhibitors of CYP3A4 or inducers of CYP3A4 is not recommended.
Sirolimus is extensively metabolized by O-demethylation and/or hydroxylation. Seven major metabolites, including hydroxyl, demethyl, and hydroxydemethyl, are identifiable in whole blood. Sirolimus is the major component in human whole blood and contributes to greater than 90% of the immunosuppressive activity.

1.2.4 Clinical efficacy in the vascular anomalies

A retrospective review was performed on six cases of complicated vascular anomaly (one

vascular tumor, 4 lymphatic malformation and 1 Klippel-Trenaunay syndrome) treated with sirolimus after failing multiple other treatments (the total number of prior interventions ranged from 2 to 5, but all patients continued to experience significant morbidities with risk of mortality) [18]. All patients were treated with the liquid formulation of sirolimus. Initial dosing was 0.8 mg/m^2 per dose, administered twice daily. Dosing adjustments were made in order to maintain a drug level of 10–15 ng/ml.
All six patients had significant responses to sirolimus: One patient with a rare but aggressive vascular tumor with lymphatic anomalies had rapid improvement in platelet count and fibrinogen level, and clinical improvement of her high-output heart failure. Four children with diffuse microcystic lymphatic malformations, causing chylous pleural effusions had rapid improvement; chest tube output decreased substantially over a short period of time, such that all of them were able to have their chest tubes removed. The patient with Klippel-Trenaunay syndrome could be released from his postoperative drainage.

Another small series of six patients with vascular anomalies (two Kaposiform hemangioendothelioma, two lymphaticovenous malformations and three lymphatic malformations) showed the same positive response to sirolimus medication [28]. Several other case reports on the efficacy of sirolimus for the treatment of several complicated vascular anomalies have been published: two on Proteus syndrome and Bannayan-Riley-Ruvacalba syndrome (BRRS) (resulting from a PTEN mutation)[19,29], two on Kaposiform hemangioendothelioma [30,31], one on BRBN [20] and another one on venolymphatic malformation [32].

We demonstrated that venous malformations (VM) are caused by activating mutations in TIE2. They also activate the PI3KAKTmTOR signaling pathway. After generating a VM mouse model, we showed that sirolimus reduced the size of the lesions in this model [33]. We subsequently initiated a pilot study on six patients with debilitating venous or complex-combined malformations that were refractory to standard care [33]. We showed that sirolimus reduced pain, functional impairment, bleeding, intravascular coagulopathy and lesion size in patients.

1.2.5 Clinical safety and tolerability

There have been reports of impaired or delayed wound healing in patients receiving sirolimus, including lymphocele and wound dehiscence. Patients with a body mass index (BMI) greater than 30 kg/m^2 may be at increased risk of abnormal wound healing based on data from the medical literature. There have also been reports of fluid accumulation, including peripheral oedema, lymphoedema, pleural effusion and pericardial effusions (including haemodynamically significant effusions in children and adults) in patients receiving sirolimus. The use of sirolimus in renal transplant patients was associated with increased serum cholesterol and triglycerides that may require treatment. Patients administered sirolimus should be monitored for hyperlipidemia using laboratory tests and if hyperlipidemia is detected, subsequent interventions such as diet, exercise, and lipid-lowering agents should be initiated. The risk/benefit should be considered in patients with established hyperlipidemia before initiating an immunosuppressive regimen, including sirolimus. Similarly the risk/benefit of continued sirolimus therapy should be re-evaluated in patients with severe refractory hyperlipidemia.

In the series of Hammill et al [18], patients with lymphatic and/or vascular malformations presented hypercholesterolemia (Grade I), mouth sore (Grade II), headache (Grade II), increased AST (Grade II), mucositis (Grade III), increased ALT/AST (Grade III), neutropenia (Grade III). Increased AST and mucositis resolved at lower dose. For the other side effects, a therapy has been ongoing with complete resolution of the symptoms.

2. TRIAL JUSTIFICATION:

Up to now, therapeutic options for extensive complex slow-flow vascular malformations such as microcystic lymphatic malformations, generalized lymphatic anomalies (GLA), extensive venous, combined lymphaticovenous malformations as well as Proteus syndrome were limited. Recent preclinical studies have shown the important role of the PI3K/AKT/mTOR pathway on the development and the lymphaticovascular organization, suggesting that sirolimus could appear as a promising therapeutic agent for patients with complex vascular malformations. No established standard of care or data from prospective clinical trials currently exist. The efficacy of sirolimus has only been demonstrated in case reports or in small series of selected patients.

Because of the limited number of reported patients treated with sirolimus for vascular anomalies, a larger study is now warranted in a wider range of clinical phenotypes and more important number of patients, in order to be able to perform statistical analysis for various signs and symptoms, to evaluate long-term outcome and give an algorithm for the management of these complicated vascular malformations.

3. TRIAL OBJECTIVES:

3.1 Purpose:

The aim of this clinical study is to prospectively evaluate the efficacy and safety of sirolimus, in the treatment of children and adults with vascular anomalies for which conventional therapies such as classical medical treatment, surgical resection and/or sclerotherapy are ineffective or associated with high risk of severe complications.

3.2 Primary objectives:

The primary objectives of this prospective multicentric study are

- ➢ to assess the potential statistical efficacy of the drug
  - whether sirolimus treatment can alleviate signs and symptoms caused by these complex vascular anomalies that are refractory to standard care
  - whether patients with this rare disorder will see their quality of life improved.
  - whether this treatment can statistically reduce the anemia and coagulation abnormalities observed in some patients with venous anomalies.

3.3 Secondary objectives:

- ➢ to confirm the safety of this treatment
- ➢ to see which specific vascular anomalies would benefit the most, and thus indicated for sirolimus in the future
- ➢ to assess whether sirolimus could reduce volume of the malformation (on MRI) on a long-term follow-up

4. TRIAL DESIGN:

This is a single arm, multicenter, interventional prospective phase III study, off-label drug trial, using sirolimus. The study is a crossover trial with control being the chronic history of the patient before the study. The study includes maximum 250 patients aged from 3 months to 70 years suffering from lymphatic, venous and/or complex vascular anomalies that are refractory to standard care. Patients will receive sirolimus for a period of 2 years and will be followed for a period of 5 years.

4.1 Screening phase

Before initiation of therapy with sirolimus, patients will be examined both by the physician specialized in vascular anomalies for their lesion and by the oncologist (adult or pediatric) for monitoring of the drug. The following issues will be done at the consultation before entering the trial:

- General clinical examination and notification of all signs and symptoms related to the malformation (see Appendix 4: Questionnaire- CRF (REDCap):
  - History of the disorder with description of previous treatments done (Patient Data Baseline protocol on REDCap)
  - Precise diagnosis, location and symptoms caused by the malformation (Clinical Parameters on REDCap)
    - Pain: intensity evaluated by visual analog scale (VAS ranging from 0 to 10), frequency and duration
    - Functional impairment
    - Esthetic impairment
  - Clinical examination: (Clinical Parameters on REDCap)
    - Location of the malformation
    - Color, consistency
    - Size
    - Tissue affected
  - Consequence on daily life and psychological impact by filling a quality of life questionnaire (adapted to MOS SF-36 Survey)
- Clinical pictures of visible lesions
- Notification of the concomitant current medications
- Revision of inclusion and exclusion criteria
- Signature of informed consent form
- Blood test (D-dimers, fibrinogen, blood count, coagulation test, hepatic, renal and medullary functions)
- Beta HCG for potentially pregnant women
- MRI (T1 and T2-weighted imaging with fat saturation in two orthogonal planes)
- If necessary, nasopharyngeal fibroscopy with pictures in case of cervico-facial internal lesions
- When possible, a 10ml EDTA- blood sample and a tissue biopsy of the affected area will be collected. These will be used to help study eventual differences in response to treatment. The tissue samples are collected in "RNA later" and shipped to the Laboratory of Human Molecular Genetics (Prof Miikka Vikkula), de Duve Institut, UCL, Brussels, Belgium. Sample collection is not mandatory for inclusion in the trial, but it is highly recommended. An additional informed consent will be signed.

-

4.2 Enrollment

The patients will be enrolled into the treatment period after informed consent (see Appendix 5) and screening phase completion in each multidisciplinary Center specialized in vascular anomalies.

4.3 Treatment phase/duration of treatment

Route of administration and dosage: (Introduction of sirolimus treatment on REDCap)
For children : first dose of sirolimus is 0,8 mg/m^2 of body surface, twice daily, either as a coated tablet or as an oral solution (recommended for children younger than 12 years or people who have difficulties to swallow such tablets).
For adult: first dose of sirolimus is 2 mg/day, as a coated tablet.

For both children and adult, serum level of sirolimus will be performed after one month of treatment consultation) and will be correlated to clinical efficacy and biological and/or clinical parameters. Serum levels will be helpful to determine whether the dose could be increased in patients who tolerate well this drug but in whom no benefit has been seen after one month. Serum levels will also be helpful to adapt sirolimus dosage related to major side effects and in case of concomitant drugs with potential interaction. Therapeutic serum levels of sirolimus should not exceed 15 ng/ml. This treatment will be continued for 2 years unless patients do not benefit from the medication 3 months after initiation, or have major side-effect of the medication.

Response assessment: (Follow-up of sirolimus treatment on REDCap):
After one month of treatment, patient will be evaluated for their vascular malformation and the medication. This will be repeated once a month for the first 3 months and then every 3 months.
At each consultation,

- ➢ Evaluation of the malformation will consist of:
  - o Notification of all signs and symptoms caused by the malformation
    - Pain: intensity evaluated by visual analog scale (VAS ranging from 0 to 10), frequency and duration
    - Functional impairment
    - Esthetic impairment
  - o Clinical examination:
    - Color, palpation
    - Size
  - o Filling in the quality of life questionnaire (adapted to MOS SF-36 Survey)
  - o Notification of the self perception of improved quality of life
- Clinical photographs of the lesions
- Notification of the concomitant current medication
- Notification of all the possible side effects
- Biological evaluation
  - o Sirolimus serum level after one month, in case of any grade 3-4 side-effect and/ or potential drug interaction.
  - o D-Dimer and fibrinogen level

- o Biological parameters such as hemogram, glycemia, cholesterolemia, triglyceridemia, renal function and hepatic function.

At 12 months of treatment, a volumetric MRI and a nasopharyngeal fibroscopy (if necessary) will be performed.

Side effects assessment:
Side effects will be evaluated according to the CTCAE version 4 by clinical and biological analysis. Follow-up of safety and criteria of discontinuation will be developed during the treatment. Conduct of the trial will be reassessed during the trial.

4.4 End of treatment

If a complete response (CR) is observed before the 24 planned months, all the study drugs will be stopped and the patient will be followed-up during 5 years.

The patient will be removed from the treatment in case of:
- Unacceptable toxicity
- Refusal to continue by the parents or the patient
- No clinical or radiological benefit after 3-month treatment

The patient will be removed from the protocol in case of:
- Loss of medical follow-up
- Withdrawal of consent
- 5 years of follow-up
- Death

5. POPULATION – INCLUSION AND EXCLUSION CRITERIA:

Inclusion criteria:

Inclusion is limited to children and adults from 3 months to 70 years of age:

- Patients with complex vascular anomalies that are refractory to standard care such as medical treatment, surgical resection and/or sclerotherapy/embolization (ineffective or accompanied by major complications)
- Patients must have adequate bone marrow function: Hemoglobine> 10,0 g/dl, neutrophils >1500/mm³ and platelets > 100.000/mm³
- Patients must have the following laboratory values:
    - o Total serum bilirubin ≤ 1.5 x ULN (or totally bilirubin ≤3 x ULN with direct bilirubin ≤ 1.5 x ULN in patients with well documented Gilbert Syndrome)
    - o Serum alanine aminotransferase (ALT) and aspartate aminotransferase (AST) ≤ 3 x ULN (or <5.0 x ULN if hepatic metastases are present)
    - o Serum creatinine ≤1.5 x ULN. If the serum creatinine is ≥ 1.5 x ULN, then a 24-hour Creatinine Clearance must be conducted and the result must be ≥ 60 mL/min.
- Karnofsky score > 50
- Patients (or legal guardians for children) have to be able to sign the informed consent
- Women of reproductive age have to be informed that contraceptive methods are mandatory during the study time

Exclusion criteria:

Any of the following concurrent severe and/or uncontrolled medical conditions, which could compromise participation in the study or interfere with the study results:

- Impaired cardiac function or clinically significant cardiac diseases, including unstable angina pectoris, ventricular arrhythmia, valvular disease with documented compromise in cardiac function, myocardial infarction within the last 6 months, documented by persistent elevated cardiac enzymes or persistent regional wall abnormalities on assessment of LVEF function, history of documented congestive heart failure (New York Heart Association functional classification III-IV), documented cardiomyopathy, family history of congenital long or short QT, or known history of QT/QTc prolongation of Torsades de Pointes (TdP)
- Impairment of Gastro-Intestinal (GI) function or GI disease that may significantly alter the absorption of sirolimus (e.g., ulcerative diseases, uncontrolled nausea, vomiting, diarrhea ≥ Grade 2, malabsorption syndrome, or small bowel resection)
- Known hypersensitivity to drugs or metabolites from similar classes as study treatment.
- Patient has other concurrent severe and /or uncontrolled medical condition that would,in the investigator's judgment, contraindicated participation in the clinical study (e.g. acute or chronic pancreatitis, liver cirrhosis, active chronic hepatitis, severely impaired lung function with a spirometry ≤ 50% of the normal predicted value and/or O_2 saturation ≤ 88% at rest, etc.)
- Recent history of primary malignancy ≤ 5 years, including history of non-melanoma skin cancer, but with with exception of carcinoma *in situ* of cervix.
- Immunocompromised patients, including known seropositivity for HIV
- Pregnant or lactating women
- Prior treatment with PI3K and/or mTOR inhibitors

6. STUDY DRUG:

6.1 Allocation to treatment

The principal investigators in each Center will enroll patients in the study.
During a follow-up visit, after verification of the inclusion and exclusion criteria, the investigator will offer the patient to participate in the study. After the patient has signed the informed consent form, the oncologist will work out the treatment plan.
The informed consent form will be place in the investigator's folder and a copy will be scanned in the medical record of the patient.

Principal investigators:
Cliniques universitaires St Luc, B-1200 Brussels, Belgium
Professor Laurence M. Boon, MD, PhD and Doctor Emmanuel Seront, MD, PhD
Center for Vascular Anomalies
Cliniques universitaires Saint-Luc,
Brussels, Belgium

Professor Prof. Dr. Jochen Rößler
Leitender Oberarzt (Funk 12-4514)

Pädiatrische Hämatologie und Onkologie
Zentrum für Kinder- und Jugendmedizin
UNIVERSITÄTSKLINIKUM FREIBURG
Mathildenstr. 1, 79106 Freiburg, Germany

Dr Anne Dompmartin-Blanchère, MD, PhD
Pôle Médecine d'Organes et Cancérologie Médicale Service de Dermatologie CHU Clemenceau 14033 CAEN Cedex 9 02 31 27 25 10, France

Dr Samira Batul Syed
MBBS DCH DCCH RCPEd RCGP FCM BTEC Adv LASER DPD (2012)
Lead for the Staff and Associate Specialists Group at GOSH Associate Specialist in Paediatrics.(Dermatology)
Skin laser/Birthmark/Vascular Anomalies Unit.
London, Great Britain

Dr Eulalia Baselga
Cap Clínic, Unitat de Dermatologia Pediàtrica
Professora Associada
Departament de Dermatologia
Hospital de la Santa Creu i Sant Pau.
Universitat Autònoma de Barcelona
Mas Casanovas 90
08041 Barcelona, Spain

Dr Maya El Hachem
Director, Dermatology Unit
Coordinator of the vascular anomalies activities
Children's Hospital Bambino Gesù, IRCCS
Piazza San Onofrio, 4
00165, Rome- Italy
President of the Italian Society for the Study of Vascular Anomalies (SISAV)

Dr Päivi Salminen,MD
Consultant Paediatric Surgeon
Division of Paediatric Surgery
Children´s Hospital Helsinki
Cordinator of Vascular Anomaly Team
Helsinki University Hospital
Stenbäckinkatu 11
00290 Helsinki, Finland

Dr Annouk Bisdorff Bresson
Hôpital Lariboisiere
Service de Neuroradiologie de Pr Houdart
Consultation des Angiomes
2, rue Ambroise Pare
75010 Paris, France

Prof Isabelle Quere
Service de Médecine Interne et Maladies Vasculaires
EA2992 - Université Montpellier I
CHU Saint Eloi
80 Avenue Auguste Fliche
304295 Montpellier Cedex 5, France
Tel: 0033-467 33 70 25
Fax: 0033-467 33 70 23
Email: i-quere@chu-montpellier.fr

Prof Alan Irvine
Pediatric Dermatology
Our Lady's Children's Hospital
Crumlin
Dublin 12, Ireland
Tel: 00353 1 416 21 03

6.2 Study drug - sirolimus

Marketing Authorization holder: Pfizer Limited
Marketing Authorization number: EU/01/171/007-8
EV Substance Code: SUB10537MIG
ATC code: L04AA10

6.2.1 Dosing regimen

Sirolimus is for oral use only. It is available either as coated tablets of 0,5 mg, 1 mg or 2 mg or as oral solution of 1 mg/ml (recommended for children younger than 12 years or people who have difficulties to swallow tablets).
Starting dose in adult population is 2 mg/day.
Starting dose in pediatric population is 0,8 mg/m^2/day given twice a day.

6.2.2 Preparation of the study drug

Sirolimus will be prescribed by the referred oncologist. Pfizer will deliver the medication to each PI hospital's pharmacy. Medication labels must comply with the legal requirements of each country and printed in the local language. They will supply no information about the patient. The storage conditions for study drug will be described on the medication label. Sirolimus has to be taken orally each day continuously at the same hour, 30 minutes before a meal.

6.2.3 Special considerations

If vomiting occurs during the course of treatment or if the patient forgets to take a dose, no re-dosing is allowed before the next scheduled dose.

Patients must avoid consumption of vitamins or herbals supplements identified as potential CYP3A4 inhibitors and inducers (please see also Appendix 1), Seville oranges, grapefruit or grapefruit juice, grapefruit hybrids, pomelos and exotic citrus fruits from 7 days prior to the first dose of study medication and during the entire study treatment period due to potential CYP3A4 interaction with the study medication. Orange juice is allowed.

If a patient needs a surgical procedure, sirolimus has to be stopped 5 days before and 1 month after in order to avoid delay in wound healing.

6.2.4 Known undesirable effects of sirolimus

Adverse events most frequently observed with sirolimus are stomatitis / oral mucositis, fatigue, headache, nausea and diarrhea. Overall, the most frequently observed laboratory abnormalities include neutropenia, thrombocytopenia, hypercholesterolemia, and/or hypertriglyceridemia. The majority of these AEs are of mild to moderate severity and rarely require a drug interruption (CTC Grade 1-2).

6.3 Dose modifications and interruption and treating adverse events

6.3.1 Guidelines for continuation of treatment

Guidelines for continuation of treatment after toxicity are listed in Table 1.
If a patient develops an undercurrent illness or other problem considered unrelated to sirolimus that requires interruption of treatment, therapy may be resumed when all symptoms are resolved or returned to baseline. Sirolimus can be discontinued during maximum 3 weeks and can be reintroduced after adverse event recovery.

To begin a new cycle of treatment with sirolimus:

- ANC must be ≥ 1500/mm3
- Platelet count must be ≥ 100000/mm3
- All non-hematologic toxicities must be ≤ grade 1 or returned to baseline
- Absence of any discontinuation criteria

Table 1 Sirolimus recommended dose modifications and management of toxicities

In any grade 3-4 toxicity, serum sirolimus dosage should be performed to exclude supratherapeutic levels of sirolimus that could be induced for example by newly administered drug interactions.

Worst toxicity (CTCAE Grade)**	Recommended Dose Modifications & Management of Toxicities
**NONE**	
**No toxicity**	**Maintain dose level**
**HEMATOLOGICAL**	
**Neutropenia (ANC)**	
Grade 1 (ANC < LLN - 1.5 x 10^9/L)<br>Grade 2 (ANC < 1.5 - 1.0 x 10^9/L)	Maintain dose level
Grade 3 (ANC < 1.0 - 0.5 x 10^9/L)	First occurrence:<br>Omit dose until resolved to ≤ Grade 1 then :<br>• If resolved in ≤ 7 days, then maintain dose level<br>• If resolved in > 7 days, then ↓ 1 dose level<br>Second occurrence:<br>Omit dose and discontinue patient from study treatment
Grade 4 (ANC < 0.5 x 10^9/L)	Omit dose and discontinue patient from study treatment
**Febrile neutropenia**<br>*(Note: Grades 1 and 2 do not exist with CTCAE Version 4.03)*	
Grade 3 (ANC < 1.0 x 10^9/L) with fever >38.5°C and/or documented infection.	First occurrence:<br>Omit dose until ANC >1.0x10^9/L then :<br>• If resolved in ≤ 7 days, then maintain dose level<br>• If resolved in > 7 days, then ↓ 1 dose level<br>Second occurrence:<br>Omit dose and discontinue patient from study treatment
Grade 4 (life-threatening consequences or urgent intervention required)	Discontinue patient from study treatment
**Thrombocytopenia**	
Grade 1 (PLT < LLN - 75 x 10^9/L)	Maintain dose level
Grade 2 (PLT < 75 - 50 x 10^9/L)	First occurrence:<br>Omit dose until resolved to ≤ Grade 1, then:<br>• If resolved in ≤ 7 days, then maintain dose level<br>• If resolved in > 7 days, then ↓ 1 dose level<br>Second occurrence:<br>Omit dose and discontinue patient from study treatment
Grade 3 ((PLT < 50-25 x 10^9/L) and<br>Grade 4 (PLT < 25 x 10^9/L)	Omit dose and discontinue patient from study treatment
**HEPATIC**	
**Bilirubin****<br>(for patients with Gilbert Syndrome these dose modifications apply to changes in direct bilirubin only)	
Grade 1 (> ULN - 1.5 x ULN)	Maintain dose level

Worst toxicity (CTCAE Grade)**	Recommended Dose Modifications & Management of Toxicities
Grade 2 (> 1.5 - 3.0 x ULN), with ALT or AST ≤ 3.0 x ULN	Omit dose and monitor LFTs* weekly until resolved to ≤ Grade 1, then:<br>• If resolved in ≤ 7 days, then maintain dose level<br>• If resolved in > 7 days, then ↓ 1 dose level
Grade 3 (> 3.0 - 10.0 x ULN), with ALT or AST ≤ 3.0 x ULN	Omit dose and monitor LFTs* weekly until resolved to ≤ Grade 1, then:<br>• If resolved in ≤ 7 days, ↓ 1 dose level<br>• If resolved in > 7 days discontinue patient from study treatment**<br>Continue to monitor LFTs* every other week or more frequently if clinically indicated until the end of treatment with study medication.<br>Second occurrence:<br>Omit dose and discontinue patient from study treatment
Grade 4 (> 10.0 x ULN)	Omit dose and discontinue patient from sirolimus**
**AST or ALT** without concurrent bilirubin elevation**	
Grade 1 (> ULN - 3.0 x ULN)<br>Grade 2 (> 3.0 - 5.0 x ULN) if not increased from baseline	Maintain dose level
Grade 2 (> 3.0 - 5.0 x ULN) if increased from baseline	Omit dose and monitor LFTs* weekly until resolved to ≤ grade 1, then<br>• If resolved in ≤ 7 days, then maintain dose level<br>• If resolved in > 7 days, then ↓ 1 dose level
Grade 3 (> 5.0 - 20.0 x ULN)	Omit dose and monitor LFTs* weekly until resolved to ≤ Grade 1, then ↓ 1 dose level<br>Continue to monitor LFTs* every other week or more frequently if clinically indicated until the end of treatment with study medication.<br>Second occurrence:<br>Omit dose and discontinue patient from study treatment
Grade 4 (> 20.0 x ULN)	Omit dose and discontinue patient from sirolimus**
**AST or ALT with concurrent bilirubin elevation (Hy's Law)**	
AST/ALT > 3 x ULN and total bilirubin > 2 x ULN<br>with no evidence of obstruction (such as elevated ALP, malignancy, impaired glucuronidation (Gilbert syndrome) or pharmacologic factors), with no other explanation (eg. viral, alcoholic or autoimmune hepatitis, hepatobiliary disorders, cardiovasular causes, concomitant medications)	• Discontinue** the patient from sirolimus and report as SAE.<br>• Monitor patient, including LFTs*, weekly or more frequently if clinically indicated until resolved to ≤ grade 1 or stabilization.
* LFTs include: albumin, ALT, AST, total bilirubin (fractionated if total bilirubin > 2.0 x ULN), AP (fractionated if AP is grade 2 or higher) and GGT). For patients with Gilbert Syndrome: total and direct bilirubin must be monitored, intensified monitoring applies to changes in direct bilirubin only.<br>** Patients who discontinue study treatment should be monitored weekly, including LFTs or more frequently if clinically indicated until resolved to ≤ grade 1 or stabilization (no CTCAE grade change over 4 weeks).	
**ENDOCRINE/METABOLIC**	
**Fasting Plasma Glucose (FPG)**	
Grade 1 (> ULN - 160 mg/dL) [> ULN - 8.9 mmol/L]	Maintain dose level, check FPG every week for 8 weeks, then continue checking every 2 weeks until resolved

Worst toxicity (CTCAE Grade)**	Recommended Dose Modifications & Management of Toxicities
Grade 2 (>160 - 250 mg/dL) [> 8.9 - 13.9 mmol/L]	**First Occurrence:**<br>Maintain dose, re-check FPG within 24hours, if no worse than Grade 2.<br>If FPG does not resolve to ≤ Grade 1 within 14 days after initiation/ intensifying anti-diabetic treatment:<br>• omit sirolimus<br>• monitor FPG at least weekly until FPG resolves to ≤ Grade 1<br>• then re-start sirolimus and ↓ 1 dose level<br>• check FPG weekly for 8 weeks, then continue checking every 2 weeks until resolved
	**Second Occurrence despite initiation and intensifying antidiabetic treatment :**<br>• omit sirolimus and discontinue patient from sirolimus treatment.<br>• monitor FPG at least twice weekly until FPG resolves to ≤ Grade 1<br>• check FPG weekly for 8 weeks, then continue checking every 2 weeks
Grade 3 (> 250 - 500 mg/dL) [> 13.9 - 27.8 mmol/L]	Immediately omit sirolimus and manage patient according to local institutional guidelines with initiation/intensification antidiabetic treatment.<br>• monitor FPG at least twice weekly until FPG resolves to ≤ Grade 1<br>• then re-start sirolimus and ↓ 1 dose level<br>• check FPG weekly for 8 weeks, then continue checking every 2 weeks until resolved<br>Second occurrence:<br>Omit dose and discontinue patient from study treatment
Grade 4 (> 500 mg/dL) [≥ 27.8 mmol/L]	Immediately omit sirolimus and manage patient according to local institutional guidelines.<br>• discontinue patient from sirolimus<br>• monitor FPG at least twice weekly until FPG resolves to ≤ Grade 1
**CARDIAC**	
**Cardiac - QTc prolongation**	
Grade 1 (QTcF 450-480msec)<br>Grade 2 (QTcF 481-500msec)	Maintain dose level
Grade 3<br>(QTcF > 500 ms on at least 2 separate ECGs)	**First Occurrence:**<br>• omit sirolimus until recovery to Grade ≤ 1 (< 480 ms)<br>• call the study's central ECG review laboratory immediately and request an immediate manual read of the ECG<br>• check patient's serum potassium, calcium, phosphorus and magnesium, and if below lower limit of normal, correct to within normal limits<br>• review concomitant medication usage<br>• repeat ECG within one hour of the first QTcF of > 500 ms<br>• if QTcF remains > 500 ms, repeat ECG as clinically indicated, but at least once a day until the QTcF returns to < 480 ms.<br>• once QTcF prolongation has resolved, sirolimus may be restarted at a one lower dose level<br>• check ECG 1 week after restart of sirolimus treatment<br>**Second Occurrence:**<br>• discontinue patient from sirolimus<br><br>**Note:** If the ECG report shows a QTcF > 500 msec, contact the patient and instruct her to return for a repeat ECG as soon as possible.

Worst toxicity (CTCAE Grade)**	Recommended Dose Modifications & Management of Toxicities
Grade 4<br>(QTcF>500msec or >60msec change from baseline and Porsade de pointes or polymorphic ventricular tachycardia or signs/symptoms of serious arrhythmia)	Discontinue patient from treatment
**OTHER**	
**Rash**	
Grade 1	Maintain dose level. Consider to initiate appropriate skin toxicity therapy (such as antihistamines, topical corticosteroids)
Grade 2	Maintain dose level. Initiate/intensify appropriate skin toxicity therapy (such as antihistamines, topical corticosteroids)
Grade 3	Omit dose until resolved to CTCAE Grade ≤ 1, then ↓ 1 dose level in association with appropriate skin toxicity therapy.<br>Second occurrence: discontinue patient from sirolimus.
Grade 4	Omit dose and discontinue patient from sirolimus
**Fatigue (asthenia)**	
Grade 1 or 2	In any case, check hemogram, ionogram and thyroid tests.<br>Maintain dose level.
Grade 3	In any case, check hemogram, ionogram and thyroid tests.<br>Omit dose until resolved to ≤ Grade 1 and then ↓ 1 dose level. After resolution, sirolimus can be re-increased progressively with alternating dosage (2mg/1mg) during 1-2 week and then to 2 mg daily if well tolerated.
Grade 4	Omit dose and discontinue patient from sirolimus
**Diarrhea**	
Grade 1	Maintain dose level<br>• Provide dietetic recommendations and consider loperamide treatment
Grade 2	Decrease the dosage with alternating doses (2mg/1mg) until resolved to ≤ 1 grade .<br>• Provide dietetic recommendations<br>• Initiate/intensify loperamide treatment<br>• Consider prophylactic loperamide with the next dose<br>• If diarrhea can not return ≤ 1 despite optimal anti-diarrhea management and alternating dosage, omit dose until resolved to ≤ grade 1 and ↓ 1 dose level. After resolution, sirolimus can be reintroduced progressively with alternating dosage (2mg/1mg) during 1-2 week and then to 2 mg daily if well tolerated.
Grade 3	**First Occurrence:**<br>Omit dose until resolved to CTCAE Grade ≤ 1, then:<br>• Manage diarrhea with loperamide intensification<br>• If resolved to Grade ≤ 1,↓ 1 dose level<br>• Consider prophylactic loperamide with the next dose<br>• Eventually, after complete resolution, sirolimus can be re-introduced progressively with alternating dosage (2mg/1mg) during 1-2 week and then to 2 mg daily if well tolerated.<br>**Second Occurrence:**<br>Omit dose and discontinue patient from study treatment
Grade 4	Discontinue treatment

6.3.2 Management of stomatitis/oral mucositis/mouth ulcers

Stomatitis/oral mucositis/mouth ulcers due to sirolimus should be treated using local

supportive care. Please note that investigators in earlier trials have described the oral toxicities associated with sirolimus as mouth ulcers, rather than mucositis or stomatitis. If your examination reveals mouth ulcers rather than a more general inflammation of the mouth, please classify the adverse event as such.

1. For mild toxicity (Grade 1), use conservative measures such as non-alcoholic mouth wash or salt water (0.9%) mouth wash several times a day until resolution.
2. For more severe toxicity (Grade 2 in which case patients have pain but are able to maintain adequate oral alimentation, or Grade 3 in which case patients cannot maintain adequate oral alimentation), the suggested treatments are topical analgesic mouth treatments including lidocaine, natrium bicarbonate and nystatine.
3. Agents containing hydrogen peroxide, iodine, and thyme derivatives may tend to worsen mouth ulcers. It is preferable to avoid these agents.
4. Antifungal agents must be avoided unless a fungal infection is diagnosed. In particular, systemic imidazole antifungal agents (ketoconazole, fluconazole, itraconazole, etc.) should be avoided in all patients due to their strong inhibition of sirolimus metabolism, thereby leading to higher sirolimus exposures. If used, serum sirolimus level should be carefully monitored. Therefore, topical antifungal agents are preferred if an infection is diagnosed. Similarly, antiviral agents such as acyclovir should be avoided unless a viral infection is diagnosed.

In case of grade 2, sirolimus dose can be decreased either to 1mg or to an alternating dosage 2mg/1mg until resolved to grade ≤ 1 resolution and then progressively re-increased to 2mg/day.

In case of grade 3, sirolimus dose has to be omitted until grade ≤ 1 resolution. Sirolimus can then be reintroduced progressively with alternating dosage (2mg/1mg) during 1-2 week and then to 2 mg daily if well tolerated.

Note: Stomatitis/oral mucositis should be appropriately graded using the functional grading given on the CTC for adverse events, version 4.0.

6.3.3 Management of hyperlipidemia and hyperglycemia

Treatment of hyperlipidemia should take into account the pre-treatment status and dietary habits. Blood tests to monitor hyperlipidemia must be taken in the fasting state. Grade 2 hypercholesterolemia (> 300 mg/dL or 7.75 mmol/L) or Grade 2 hypertriglyceridemia (>2.5 x ULN) should be treated with a 3-hydroxy-3-methyl-glutaryl (HMG)-CoA reductase inhibitor (e.g., atorvastatin, pravastatin) or appropriate lipid-lowering medication, in addition to diet. Patients should be monitored clinically and through serum biochemistry for the development of rhabdomyolysis and other adverse events as required in the product label/data sheets for HMG-CoA reductase inhibitors.

Grade 3 hyperglycemia has been observed in patients receiving sirolimus therapy. In many cases in study sirolimus, the affected patients had an abnormal fasting glucose at baseline. Based on this finding, it is suggested that optimal glucose control should be achieved before starting a patient on sirolimus and should be monitored during sirolimus therapy.

6.3.4 Management of diarrhea

Table 2. CTC grading of diarrhea

Grade	Definition
Grade 1	increase of < 4 stools/day over baseline; mild increase in ostomy output compared to baseline
Grade 2	Increase of 4-6 stools/day over baseline, moderate increase in ostomy output compared to baseline
Grade 3	increase of ≥7 stools/day over baseline; incontinence; hospitalization indicated; severe increase in ostomy output compared to baseline; limiting self care ADL
Grade 4	Life threatening consequences; urgent intervention indicated

• Stop all lactose-containing products and laxative, bulk fiber nutrients.
• Assure a correct hydratation.
It is further recommended that patients will be provided with loperamide tablets (or prescription) with the start of sirolimus treatment. When provided with loperamide, it is mandatory that patients are instructed on the correct use in order to manage signs or symptoms of diarrhea at home. The standard dose is 4 mg followed by 2 mg every 4 hrs or after each unformed stool (maximum 16 mg/day). This dose may be increased in patients with mild to moderate diarrhea (Grade 1 or 2) that persists for more than 24 hours Loperamide should be discontinued after a 12 hours diarrhea-free interval. Severe diarrhea grade 3 or 4 or complicated grade 1 or 2 diarrhea may require hospitalization, and assessment of CBC, electrolytes and stool workup (e.g. cultures to exclude infectious causes) are recommended.

6.3.5 Headache

Headache is frequently observed with sirolimus but is usually well tolerated (most frequently grade 1 and 2) and well controlled by symptomatic treatments (paracetamol and/or non steroid anti-inflammatory). Work-up of headache requires standard clinical and neurological examination if needed. In case of refractory headache, sirolimus dose can be omitted and next dose level can be reduced. Severe, atypical and unusual headache requires of course radiological investigations to exclude vital events (cerebral bleeding or cerebrovascular events). History of headache is not a contraindication for sirolimus.

6.3.6 Management of non-infectious pneumonitis

Both asymptomatic radiological changes (Grade 1= radiological lung changes only) and symptomatic non-infectious pneumonitis (Grade 2 = not interfering with activities of daily living or Grade 3 = interfering with activities of daily living and oxygen indicated) have been noted in patients receiving sirolimus therapy. Non-infectious pneumonitis has been associated with sirolimus. If non-infectious pneumonitis develops, consultation with a pulmonologist is recommended. Management of non-infectious pneumonitis suspected to be associated with sirolimus and dose modification instructions are provided in Table 1-1.

Table-3 Management of non-infectious pneumonitis

Worst Grade Pneumonitis	Required Investigations	Management of Pneumonitis	Dose Adjustment
Grade 1 dose	CT scans with lung windows. Repeat at least every 3 cycles until return to normal limits	No specific therapy	Administer 100% of sirolimus
Grade 2	CT scan with lung windows and pulmonary function testing including : spirometry, DLCO, and room air O2 saturation at rest ; repeat each subsequent cycle until return to baseline. Consider bronchoscopy*	Symptomatic only. Prescribe corticosteroids if cough is troublesome	Reduce sirolimus dose until recovery to ≤ grade 1. Sirolimus may also be interrupted if symptoms are troublesome. Patients will be withdrawn from the study if they fail to recover to ≤ grade 1 within 3 weeks. Sirolimus cannot be escalated
Grade 3-4	CT scan with lung windows and pulmonary function testing including : spirometry, DLCO, and room air O2 saturation at rest ; repeat each subsequent cycle until. return to baseline Bronchoscopy and bronchoalveolar lavage is recommended	Prescribe corticosteroids if infective origin is ruled out. Taper as medically indicated	Discontinue treatment

6.4 Treating the patient and dose modifications

6.4.1 Sirolimus dosing instructions

Sirolimus prescription will be dispensed by a medical doctor at baseline and on day 1 of each cycle. Patients will be provided with an adequate supply of sirolimus for self-administration at home. The investigator should instruct the patient to take sirolimus exactly as prescribed (promote compliance). All dosages prescribed and dispensed to the patient and all dose changes during the study must be recorded on the Dosage Administration Record CRF. Thereafter, patients will be instructed to take one 2 mg (or adequate dose per body weight) tablet of sirolimus orally with a glass of water, once daily at the same time each day, continuously.

At the time of dosing, the patient should be in a fasting state or has eaten no more than a light, fat-free meal. Any dietary habits around the time of sirolimus intake should be as consistent as possible throughout the study. If vomiting occurs, no attempt should be made to replace the vomited dose unless two sirolimus tablets are clearly visible.

6.4.2 Dose modifications

For patients who are unable to tolerate the protocol-specified dosing schedule, dose adjustments are permitted in order to keep the patient on sirolimus. The guidelines set forth in Table 1 should be followed: If treatment is interrupted due to toxicity, sirolimus should not be resumed until recovery to ≤ Grade 1, then reintroduce sirolimus at the initial dose or lower dose level depending on toxicity type and Grade (Table 4). These changes must be recorded on the Dosage Administration Record CRF.

Table 4 Dose reduction steps for sirolimus

Dose level	Dose and schedule
0 (starting dose)	2 mg daily
Decrease 1 dose level	1 mg daily

If a patient has already decreased 1 dose level, no further dose reduction is permitted. Patients requiring a second dose reduction must discontinue sirolimus. The maximum allowed time of interruption of sirolimus is 3 weeks.

6.4.3 Follow-up for toxicities

Patients whose treatment is interrupted or permanently discontinued due to an adverse event or abnormal laboratory value must be followed at least once a week for 4 weeks, and subsequently at 4-week intervals, until resolution or stabilization of the event, whichever comes first. If a patient requires a dose delay of both medications > 21 days from the intended day of the next scheduled dose, then the patient must be discontinued from the study. However, the patient will continue to be followed for toxicity as previously described.

6.5 Concomitant Medication(s)

Patients must be instructed not to take any additional medications (over-the-counter or other products) during the study without prior consultation with/approval from the investigator. All medications taken within 30 days of starting study treatment should be reported on the Concomitant Medication/Significant Non-drug Therapy Prior to Start of Study Drug CRF. The investigator should instruct the patient to notify the study center about any new medications he/she takes after the start of the study treatment. All medications (other than study treatment) and significant non-drug therapies (including physical therapy and blood transfusions) administered after the patient starts treatment with study drug must be listed on the Concomitant medications/Significant non-drug therapies after start of study drug CRF.

The following concomitant treatments should be avoided unless use of the drug is essential and no substitute is available (Appendix 1, Table A):

Substrates (competitive inhibition):
- Antibiotics: clarithromycin*, erythromycin, telithromycin*
- Anti-arrhythmics: quinidine
- Benzodiazepines: alprazolam, diazepam, midazolam, triazolam

- Immune Modulators: cyclosporine, tacrolimus (FK506)
- HIV Protease Inhibitors: indinavir*, ritonavir*, saquinavir*
- Antihistamines: astemizole, chlorpheniramine
- Calcium Channel Blockers: amlodipine, diltiazem, felodipine, nifedipine, nisoldipine, nitrendipine, verapamil
- HMG-CoA Reductase Inhibitors: cerivastatin, lovastatin, simvastatin
- Miscellaneous: aripiprazole, aprepitant, buspirone, gleevec*, haloperidol, methadone, pimozide, quinine, sildenafil, tamoxifen, trazodone, vincristine

Inducers:
Carbamazepine, Phenobarbital, Phenytoin*, Rifabutin*, Rifampin*, St John's wort, Troglitazone

Inhibitors:
Amiodarone, Cimetidine, Clarithromycin, Delaviridine, Diltiazem, Erythromycin, Fluvoxamine*, Grapefruit juice, Sevilla orange, Indinavir, Itraconazole*, Ketoconazole*, Voriconazole*, Posaconazole*, Mibefradil, Nefazodone*, Nelfinavir*, Ritonavir*, Troleandomycin, Verapamil [34]

* asterisk denotes strong inhibition/ induction

If a patient enrolled in the study was previously treated with one of these listed drugs, sirolimus can be started but with a careful monitoring of tolerance profile and serum sirolimus level control (after 7 days and then monthly during treatment with these drugs).
If a patient enrolled in this study and receiving sirolimus has to be treated with one of these listed drugs, treatment with these drugs has to be shortest as possible with a close monitoring of tolerance profile and serum sirolimus level control (after 7 days and then monthly during treatment with these drugs).

Please note:
- strong inhibitor implies that it can cause ≥5-fold increase in AUC or ≥80% decrease in clearance of sensitive CYP substrates
- moderate inhibitor implies that it can cause 2 to 5-fold increase in AUC values or 50-80% decrease in clearance of sensitive CYP substrates. (Distinction is not always categorical as interaction can vary according to conditions).
- Macrolide antibiotics: Azithromycin is not a CYP3A substrate. It may therefore be employed where antibiotic therapy with a macrolide is desirable in a patient being treated with sirolimus
- In a same way, interaction with drugs increasing QT and facilitating Tosades de pointe, has to be closely assessed (Appendix 1, Table B)

Statins: Atorvastatin OR Pravastatin may be co-administered with sirolimus, since a PK interaction study has shown that there is no relevant PK interaction.
If any of these drugs are necessary, close assessment of the serum sirolimus level has to be performed after 7 days, with adequate sirolimus dosage adjustement.

6.6 Study treatment discontinuation

The term "interruption" refers to a patient stopping the study medication during the course of the study, but then re-starting it at a later time during the study.

The term “discontinuation” refers to a patient’s withdrawal from the study treatment. The reason for discontinuation from treatment will be recorded. The patient may discontinue study treatment for any of the following reasons:

- Adverse event(s) not accepted by the patient or grade 4 and not curative
- Subject withdrew consent
- Lost to follow-up
- Administrative problems
- No positive effect on the malformation
- Death

If a patient has discontinued the study treatment due to an unacceptable adverse drug reaction or an abnormal laboratory value, he/she should not have withdrawal of consent recorded as the reason for discontinuation. Instead, the reason for discontinuation must be recorded as due to drug-induced adverse event.

6.7 Premature patient withdrawal

Patients may voluntarily withdraw from the study or be taken off study at the discretion of the investigator at any time. If such withdrawal occurs, or if the patient fails to return for follow-up visits, the investigator must determine the primary reason for a patient’s withdrawal from the study and record this information on the Study Evaluation Completion CRF. As a general rule, if a patient discontinues study treatment and later is withdrawn from the study, the reasons for study evaluation completion may include the following:

- Protocol deviation
- Subject withdrew consent
- Lost to follow-up
- Administrative problems
- Death

For patients who are lost to follow-up, the investigator should show "due diligence" by documenting in the patients’ source documents, steps taken to contact the patient, e.g., dates of telephone calls, registered letters, etc.

7. VISIT SCHEDULES:

The table below lists all of the assessments and indicates with an “X” the visits when they are performed.

Visit evaluation schedule

Assessment	Baseline	Starting day	Month +1	Month +2	Month +3	Month +6	Subsequent visit every 3 months	End of Study Treatment	Follow Up: visit every 4 months
Visit	1	2	3	4	5	6	7+	Last Visit	
Day	D -14 to -1*	0	30	60	90	180	300 and beyond	<7 days from last dose	Starting 30 days after last dose
Baseline[a]	X								
Vital signs[b]	X	X	X	X	X	X	X	X	X
Physical Exam[b]	X	X	X	X	X	X	X	X	X
Karnofsky [b]	X	X	X	X	X	X	X	X	X
Hematology [c]	X		X		X	X	X (every 6 months)	X	X (every 8 months)
Coagulation Studies[d]	X		X		X	X	X (every 6 months)	X	X (every 8 months)
Serum Chemistry[e]	X		X		X	X	X (every 6 months)	X	X (every 8 months)
Serum Lipid Profile[f]	X					X	X (every 6 months)	X	X (every 8 months)
MRI[g]	X						X (annually)		
Serum pregnancy test[h]	X								
Thyroid tests I[i]	X					X	X (every 6 months)		
Adverse Events (AE) /Serious AE[k]	Continuous								
Prior/Concomitant Medications[l]	Continuous								
Sirolimus dosing[m]			X	X (in case of major side effects or potential concomitant drug interaction)					
Study completion								X	
Follow-Up Visit									X

[a] Baseline evaluations include: demography, informed consent, inclusion/exclusion criteria, relevant medical history/current medical conditions, current medications, vital signs and symptoms, laboratory tests.

[b] Vital signs, physical exam and Karnofsky will be performed at baseline (within 2 weeks prior to the first dose of study treatment), repeated monthly during the first three months and then every three months (± 7 days of scheduled visit), at the end of study treatment and in the follow-up (every four months). Significant findings from clinical signs and symptoms will be noted in the relevant medical history page or adverse events pages.

[c] Hematology includes haemoglobin, hematocrit, platelets, total white blood cell count (WBC) & differential and will be performed at baseline (within 2 weeks prior to the first dose of study treatment), after one, three and six month and then every six months (± 7 days of scheduled visit), at the end of study treatment and in the follow-up (every eight months).

[d] Coagulation tests including INR, TCA, Fibrinogen and D-Dimers evaluation will be performed at baseline (within 2 weeks prior to the first dose of study treatment), after one, three and six month and then every six months (± 7 days of scheduled visit), at the end of study treatment and in the follow-up (every eight months).

[e] Serum chemistry includes: LDH, fasting glucose, sodium, phosphorus, sodium potassium, magnesium, chloride, creatinine, blood urea nitrogen, albumin, total protein, SGOT (AST), SGPT (ALT), total bilirubin, GGT, alkaline phosphatase, urea, uric acid, and serum corrected calcium and be performed at baseline (within 2 weeks prior to the first dose of study treatment), after one, three and six month and then every six months (± 7 days of scheduled visit), at the end of study treatment and in the follow-up (every eight months).

[f] Serum Lipid profile includes: total cholesterol, triglycerides, LDL and HDL will be performed at baseline (within 2 weeks prior to the first dose of study treatment), every six months (± 7 days of scheduled visit), at the end of study treatment and in the follow-up (every eight months).

[g] An MRI of the body part affected by the malformation will be performed within 3 months prior to the first dose of study drug and then every year.

[h]Serum pregnancy test. Women of childbearing potential must have a serum pregnancy test performed ≤ 72 hours prior to the first dose of study drug.

[i]Thyroid stimulating hormone (TSH) will be analyzed at baseline (within 2 weeks) and then every 6 months during the study drug administration.

[k]Adverse events. All adverse events occurring after the start of the study even if the event is not considered to be related to the study drug must be documented on Adverse Event CRFs.

[l]Prior concomitant medications. Record all medications given within 30 days prior to administration to administration of study treatment

[m]Sirolimus serum level will be performed after one month, in case of any grade 3-4 side-effect and/ or potential drug interaction.

8. EVALUATION CRITERIA – EFFICACY AND SAFETY:

8.1 Response assessment

After one month of treatment, a general clinical examination is performed with assessment of signs and symptoms (functional, psychological and esthetic), pain (frequency, duration and intensity according to VAS), quality of life (using a quality of life questionnaire adapted to MOS SF-36 Survey), side effects and treatment compliance. Clinical photographs of the malformation will be taken, if relevant. Serum sirolimus level is assessed only after 1 month of treatment. A complete blood control will be done. This will be done by the principal investigator and the oncologist.
This assessment will be continued at a monthly base for the first 3 months and then every 3 months. At 12 months of treatment, a volumetric MRI and a nasopharyngeal fibroscopy (if necessary) will be performed.

8.2 Side effects assessment

Side effects will be evaluated according to the CTCAE version 4 at each consultation by the oncologist. Follow-up of safety and criteria of discontinuation will be developed during the treatment. Conduct of the trial will be reassessed during the trial.

9. ADVERSE EVENT REPORTING:

9.1 Adverse events

All observed adverse events regardless of treatment group or suspected causal relationship to the investigational product(s) will be reported as described in the following sections.

For all adverse events, the investigator must pursue and obtain information adequate both to determine the outcome of the adverse event and to assess whether it meets the criteria for classification as a serious adverse event (see Section 9.5) requiring immediate notification to the study coordinator (Principal Investigator). For all adverse events, sufficient information should be obtained by the investigator to determine the causality of the adverse event. The investigator is required to assess causality. For adverse events with a causal relationship to the investigational product, follow-up by the investigator is required until the event or its sequelae resolve or stabilize at a level acceptable to the investigator, and the study coordinator concurs with that assessment.

9.2 Reporting period

Serious adverse events require immediate (within 24 hours) notification to beginning from the time that the patients provides informed consent, which is obtained prior to the patient's participation in the clinical trial, i.e., prior to undergoing any trial-related procedure and/or receiving investigational product, through and including 28 calendar days after the last administration of the investigational product. Any serious adverse event occurring any time after the reporting period must be promptly reported if a causal relationship to investigational product is suspected.

Adverse events (serious and non-serious) should be recorded on the CRF from the time the patient has taken at least one dose of trial treatment through last patient visit.

9.3 Definition of an adverse event

An adverse event is any untoward medical occurrence in a clinical investigation patient administered a product or medical device; the event need not necessarily have a causal relationship with the treatment or usage. Examples of adverse events include but are not limited to:

• Abnormal test findings;
• Clinically significant symptoms and signs;
• Changes in physical examination findings;
• Hypersensitivity.

Additionally, they may include the signs or symptoms resulting from:
• Drug overdose;
• Drug withdrawal;
• Drug abuse;
• Drug misuse;
• Drug interactions;
• Drug dependency;
• Exposure in utero.

9.4 Abnormal test findings

The criteria for determining whether an abnormal objective test finding should be reported as an adverse event are as follows:

• Test result is associated with accompanying symptoms, and/or
• Test result requires additional diagnostic testing or medical/surgical intervention, and/or
• Test result leads to a change in trial dosing or discontinuation from the trial, significant additional concomitant drug treatment, or other therapy, and/or
• Test result is considered to be an adverse event by the investigator.

Merely repeating an abnormal test, in the absence of any of the above conditions, does not constitute an adverse event. Any abnormal test result that is determined to be an error does not require reporting as an adverse event.

9.5 Serious adverse events

A serious adverse event or serious adverse drug reaction is any untoward medical occurrence at any dose that:

- Results in death;
- Is life-threatening (immediate risk of death);
- Requires inpatient hospitalization or prolongation of existing hospitalization;
- Results in persistent or significant disability/incapacity;
- Results in congenital anomaly/birth defect.

Medical and scientific judgment should be exercised in determining whether an event is an important medical event. An important medical event may not be immediately life-threatening and/or result in death or hospitalization. However, if it is determined that the event may jeopardize the patient and may require intervention to prevent one of the other outcomes listed in the definition above, the important medical event should be reported as

9.6 Hospitalization

Adverse events reported from clinical trials associated with hospitalization or prolongation of hospitalization are considered serious. Any initial admission (even if less than 24 hours) to a healthcare facility meets these criteria. Admission also includes transfer within the hospital to an acute/intensive care unit (eg, from the psychiatric wing to a medical floor, medical floor to a coronary care unit, neurological floor to a tuberculosis unit).

Hospitalization does not include the following:

- Rehabilitation facilities;
- Hospice facilities;
- Respite care (eg, caregiver relief);
- Skilled nursing facilities;
- Nursing homes;
- Routine emergency room admissions;
- Same day surgeries (as outpatient/same day/ambulatory procedures).

Hospitalization or prolongation of hospitalization in the absence of a precipitating, clinical adverse event is not in itself a serious adverse event. Examples include:

- Admission for treatment of a preexisting condition not associated with the development of a new adverse event or with a worsening of the preexisting condition (eg, for work-up of persistent pre-treatment lab abnormality);
- Social admission (eg, patient has no place to sleep);
- Administrative admission (eg, for yearly physical exam);
- Protocol-specified admission during a clinical trial (eg, for a procedure required by the trial protocol);
- Optional admission not associated with a precipitating clinical adverse event (eg, for elective cosmetic surgery);
- Pre-planned treatments or surgical procedures should be noted in the baseline documentation for the entire protocol and/or for the individual patient.
- Admission exclusively for the administration of blood products.

9.7 Severity assessment (see Appendix 2)

If required on the adverse event case report forms, the investigator will use the following definitions of severity in accordance with Common Terminology Criteria for Adverse Events (CTCAE) Version 4.0 to describe the maximum intensity of the adverse event. If the event is serious, the CTCAE grade reported in the adverse event CRF must be consistent with the description of CTCAE grade included in the narrative section of the serious adverse event report.

GRADE	Clinical Description of Severity
0	No Change from Normal or Reference Range (This grade is not included in the Version 3.0 document but may be used in certain circumstances.)
1	MILD Adverse Event

2	MODERATE Adverse Event
3	SEVERE Adverse Event
4	LIFE-THREATENING OR DISABLING Adverse Event
5	DEATH RELATED TO Adverse Event

Note the distinction between the severity and the seriousness of an adverse event. A severe event is not necessarily a serious event. For example, a headache may be severe (interferes significantly with subject's usual function) but would not be classified as serious unless it met one of the criteria for serious adverse events, listed above.

9.8 Causality assessment

The investigator's assessment of causality must be provided for all adverse events (serious and non-serious). An investigator's causality assessment is the determination of whether there exists a reasonable possibility that the investigational product caused or contributed to an adverse event. If the investigator's final determination of causality is unknown and the investigator does not know whether or not investigational product caused the event, then the event will be handled as «related to investigational product» for reporting purposes. If the investigator's causality assessment is "unknown but not related to investigational product", this should be clearly documented on trial records.

In addition, if the investigator determines a serious adverse event is associated with trial procedures, the investigator must record this causal relationship in the source documents and CRF, as appropriate, and report such an assessment in accordance with the serious adverse event reporting requirements, if applicable.

9.9 Adverse event reporting requirements

Each adverse event is to be assessed to determine if it meets the criteria for serious adverse event. If a serious adverse event occurs, expedited reporting will follow local regulation, as appropriate.

All adverse events will be reported on the adverse event page(s) of the CRF. It should be noted that the form for collection of serious adverse event information is not the same as the adverse event CRF. Where the same data are collected, the forms must be completed in a consistent manner. For example, the same adverse event term should be used on both forms. Adverse events should be reported using concise medical terminology on the CRFs as well as on the form for collection of serious adverse event information.

9.10 Serious adverse event reporting requirements (see Appendix 3)

For the purpose of regulatory reporting, SPONSOR will determine the expectedness of events suspected of being related to Sirolimus based on the SmPC. Sponsor will report in an expedited manner to Regulatory Authorities and Ethics Committees concerned, suspected

unexpected serious adverse reactions (SUSARs) in accordance with Directive 2001/20/EC and the Detailed Guidance on collection, verification and presentation of adverse reaction reports arising from clinical studies on investigational products for human use (Eudralex Volume 10/CT3) and also in accordance with country-specific requirements. Sponsor shall notify the Investigator of any AE associated with the use of IP in this study that is both serious and unexpected (ie, SUSAR); The Investigator must notify his/her IRB/EC promptly of these new serious and unexpected AE(s) or significant risks to subjects.

If the serious adverse event is fatal or life-threatening, notification must be made immediately to study coordinator of the trial center (data nurse or investigator), irrespective of the extent of available adverse event information. This timeframe also applies to additional new information (follow-up) on previously forwarded serious adverse event reports.

The study coordinator (Principal Investigator) will take care of all the regulatory procedure according to the European regulation. In the rare event that the investigator does not become aware of the occurrence of a serious adverse event immediately (eg, if an outpatient trial patient initially seeks treatment elsewhere), the investigator is to report the event within 24 hours after learning of it and document the time of his/her first awareness of the adverse event. In the case of a patient death, a summary of available autopsy findings must be submitted as soon as possible to the study coordinator.

10. QUALITY CONTROLE AND QUALITY ASSURANCE:

The confidentiality of the data is guarantee by anonymous data transfer and conduct under the Belgian regulation (« Cela respectera la confidentialité médicale, conformément à la loi sur la protection des données personnelles (du 8 décembre 1992) et la loi sur la protection du patient (du 22 août 2002) »).

The patient can have access to his/her medical file upon request (loi du 22 août 2002, Art. 9 § 2: « Le patient a droit à la consultation du dossier le concernant »).

11. DATA HANDLING AND RECORD KEEPING:

11.1 Case Report Forms – Questionnaire (see Appendix 4)

As used in this protocol, the term case report form (CRF) should be understood to refer to either a paper form or an electronic data record or both, depending on the data collection method. In this trial, the CRF will be constituted by the documents of REDCap (Research Electronic Data Capture) that will be completed after each patient visit.

A CRF is required and should be completed for each included subject. The completed original CRFs are the sole property of the study coordinator and should not be made available in any form to third parties, except for authorized representatives of the study coordinator or appropriate regulatory authorities.

It is the investigator's responsibility to ensure completion and to review and approve all CRFs. CRFs must be signed by the investigator or by an authorized staff member. These signatures serve to attest that the information contained on the CRFs is true. At all times, the investigator has final personal responsibility for the accuracy and authenticity of all clinical and laboratory data entered on the CRFs. Subject source documents are the

physician's subject records maintained at the trial site. In most cases, the source documents will be the hospital's or the physician's chart. In cases where the source documents are the hospital or the physician's chart, the information collected on the CRFs must match those charts.

11.2 Record retention

To enable evaluations and/or audits from regulatory authorities or the study coordinator, the investigator agrees to keep records, including the identity of all participating subjects (sufficient information to link records, eg, CRFs and hospital records), all original signed informed consent forms, copies of all CRFs, source documents, and detailed records of treatment disposition. The records should be retained by the investigator according to ICH, local regulations, or as specified in the Clinical Study Agreement, whichever is longer.

If the investigator relocates, retires, or for any reason withdraws from the trial, the study coordinator should be prospectively notified. The trial records must be transferred to an acceptable designee, such as another investigator, another institution. The investigator must obtain the study coordinator's written permission before disposing of any records, even if retention requirements have been met.

12. ETHICS:

12.1 Institutional Review Board (IRB)/Independent Ethics Committee (IEC)

It is the responsibility of each local investigator to obtain prospective approval of the trial protocol, protocol amendment, informed consent forms, and other relevant documents, eg, advertisements, if applicable, from the IRB/IEC. All correspondence with the IRB/IEC should be retained in the Investigator File. Copies of IRB/IEC approvals should be forwarded to the study coordinator.

The only circumstance in which an amendment may be initiated prior to IRB/IEC approval is where the change is necessary to eliminate apparent immediate hazards to the subjects. In that event, the investigator must notify the IRB/IEC and the study coordinator in writing within 5 working days after the implementation.

12.2 Ethical conduct of the trial

The trial will be performed in accordance with the protocol, International Conference on Harmonization Good Clinical Practice guidelines, and applicable local regulatory requirements and laws.

12.3 Subject information and consent

The informed consent form must be agreed to by the study coordinator and the IRB/IEC and must be in compliance with ICH GCP, local regulatory requirements, and legal requirements.

The investigator must ensure that each trial subject, or his/her legally acceptable representative, is fully informed about the nature and objectives of the trial and possible risks associated with participation. The investigator will obtain written informed consent from each subject or the subject's legally acceptable representative before any study-specific activity is performed. The informed consent form used in this trial, and any changes made during the

course of the trial, must be prospectively approved by both the IRB/IEC and the study coordinator before use. The Principal Investigator will retain the original of each subject's signed consent form.

13. INSURANCE AND FUNDING:

13.1 Insurance

The experimentation is covered under the Belgian Law of May 7, 2004 by a no-fault insurance (type of coverage: liability insurance). Each Center involved in the study is responsible for its own liability.

Policy holder:
Cliniques Universitaires Saint-Luc
Avenue Hippocrate, 10
1200 Brussels

Issuer of the certificate of insurance:
Amlin Corporate Insurance, S.A.
Boulevard du Roi Albert II, 9
1210 Brussels

13.2 Funding

There was no subvention by any pharmaceutical company. Pfizer will deliver the medication and cover the cost of 2 serum level of sirolimus analyses.

14. DEFINITION OF END OF TRIAL:

End of Trial is defined as:
-The time at which it is deemed that sufficient patients have been recruited and completed the trial as stated in the regulatory application (Agence Fédérale des Médicaments et des Produits de Santé (AFMPS)) and ethics committee (CEBHF of the Cliniques Universitaires Saint-Luc). Poor recruitment (recruiting less than the anticipated number in the CTA) is not a reason for premature termination but is considered a normal conclusion to the trial.

-The last subject and the last visit (LSLV)

15. PUBLICATION OF STUDY RESULTS:

The study coordinator is responsible for publication of the results. All the investigators in the trial will be co-author. They will be listed depending on the number of patients included. PI who designed the study will be last and corresponding author.

16. REFERENCES:

1. Mulliken JB, Glowacki J. Hemangiomas and vascular malformations in infants and children: a classification based on endothelial characteristics. Plast Reconstr Surg 1982 ; 69 (3): 412 – 22.
2. Mulliken JB, Young AE. Vascular Birthmarks: Hemangiomas and Malformations. Philadelphia : WB Saunders, 1988.
3. Enjolras O, Rich é MC, Mulliken JB, Merland J. Atlas des H é mangiomes et Malformations Vasculaires Superfi cielles. Paris : McGraw - Hill, 1990.
4. Takahashi K, Mulliken JB, Kozakewich HP, Rogers RA, Folkman J , Ezekowitz RA. Cellular markers that distinguish the phases of hem-angioma during infancy and childhood. J Clin Invest 1994 ; 93 (6): 2357 – 64.
5. Enjolras O, Mulliken JB . The current management of vascular birthmarks Pediatr Dermatol 1993 ; 10 (4): 311 – 13.
6. Enjolras O, Mulliken JB. Vascular tumors and vascular malformations (new issues). Adv Dermatol 1997 ; 13 : 375 – 423.
7. Dompmartin A, Acher A, Thibon P, et al. Association of localized intravascular coagulopathy with venous malformations. Arch Dermatol 2008;144:873–877.
8. Limaye N. and al, Variable Somatic *TIE2* Mutations in Half of Sporadic Venous Malformations, Mol Syndromol. 2013 Apr; 4(4): 179–183.
9. Patel DV. Gorham's disease or massive osteolysis. Clin Med Res 2005;3:65–74.
10. Boscolo E, Coma S, Luks VL, Greene AK, Klagsbrun M, Warman ML, Bischoff J. AKT hyper-phosphorylation associated with PI3K mutations in lymphatic endothelial cells from a patient with lymphatic malformation, Angiogenesis. 2015 Apr;18(2):151-62
11. Tan WH, Baris HN, Burrows PE, et al. The spectrum of vascular anomalies in patients with PTEN mutations: Implications for diagnosis and management. J Med Genet 2007;44:594–602.
12. Reinhardt MA, Nelson SC, Sencer SF, et al. Treatment of childhood lymphangiomas with interferon-alpha. J Pediatr Hematol Oncol 1997;19:232–236.
13. Dickerhoff R, Bode VU. Cyclophosphamide in non-resectable cystic hygroma. Lancet 1990;335:1474–1475.
14. Turner C, Gross S. Treatment of recurrent suprahyoid cervicofacial lymphangioma with intravenous cyclophosphamide. Am J Pediatr Hematol Oncol 1994;16:325–328
15. Hammer FD, Boon LM, Mathurin P, Vanwijck RR. Ethanol sclerotherapy of venous malformations: evaluation of systemic ethanol contamination. J Vasc Interv Radiol 2001, 12: 595-600
16. Mitra AP, Datar RH, Cote RJ. Molecular pathways in invasive bladder cancer: new insights into mechanisms, progression, and target identification. J Clin Oncol 2006; 24: 5552-64.
17. Perry B, Banyard J, McLaughlin ER, et al. AKT1 overexpression in endothelial cells leads to the development of cutaneous vascular malformations in vivo. Arch Dermatol 2007;143:504–506.
18. Hammill AM, Wentzel M, Gupta A, Nelson S, Lucky A, Elluru R, Dasgupta R, Azizkhan RG, Adams DM, Sirolimus for the treatment of complicated vascular anomalies in children, Pediatr Blood Cancer. 2011 Dec 1;57(6):1018-24
19. Marsh DJ, Trahair TN, Martin JL, et al. Rapamycin treatment for a child with germline PTEN mutation. Nat Clin Pract Oncol 2008;5:357–361.
20. Yuksekkaya H, Ozbek O, Keser M, Toy H., Blue rubber bleb nevus syndrome: successful treatment with sirolimus, Pediatrics. 2012 Apr;129(4):e1080-4
21. Falger JC, Mueller T, Arbeiter K, et al. Conversion from calcineurin inhibitor to sirolimus in pediatric chronic allograft nephropathy. Pediatr Transplant 2006;10:565–569.

22. Sindhi R, Seward J, Mazariegos G, et al. Replacing calcineurin inhibitors with mTOR inhibitors in children. Pediatr Transplant 2005;9:391–397.
23. Schachter AD, Benfield MR, Wyatt RJ, et al. Sirolimus pharmacokinetics in pediatric renal transplant recipients receiving calcineurin inhibitor co-therapy. Pediatr Transplant 2006;10:914–919.
24. Baselga J, Campone M, Piccart M, et al. **Everolimus** in postmenopausal hormone-receptor-positive advanced **breast cancer**. N Engl J Med. 2012 Feb 9;366(6):520-9.
25. Motzer RJ, Escudier B, Oudard S et al. Efficacy of **everolimus** in advanced **renal** cell carcinoma: a double-blind, randomised, placebo-controlled phase III trial. Lancet. 2008 Aug 9;372(9637):449-56.
26. Hudes G, Carducci M, Tomczak P et al. **Temsirolimus**, interferon alfa, or both for advanced **renal**-cell carcinoma. N Engl J Med. 2007 May 31;356(22):2271-81.
27. Yao JC, Shah MH, Ito T et al. **Everolimus** for advanced **pancreatic** neuroendocrine tumors. N Engl J Med. 2011 Feb 10;364(6):514-23.
28. **Lackner** H, Karastaneva A, Schwinger W, Benesch M, Sovinz P, Seidel M, Sperl D, Lanz S, Haxhija E, Reiterer F, Sorantin E, Urban CE. Eur J Pediatr. 2015 Jun 4
29. Iacobas I, Burrows PE, Adams DM, Sutton VR, Hollier LH, Chintagumpala MM ; Oral rapamycin in the treatment of patients with hamartoma syndromes and PTEN mutation. Pediatr Blood Cancer 2011 Aug;57(2):321-3
30. Blatt J, Stavas J, Moats-Staats B, Woosley J, Morrell DS. Pediatr Blood Cancer. 2010 Dec 15;55(7).
31. Wang Z, Li K, Yao W, Dong K, Xiao X, Zheng S. Pediatr Blood Cancer. 2015 Jul;62(7):1291-3
32. **Kim** D, **Benjamin** L, Wysong A, Hovsepian D, Teng J. Dermatol Ther. 2015 Jul;28(4):218-21.
33. Boscolo E, Limaye N, Huang L, Kang KT, Soblet J, Uebelhoer M, Mendola A, Natynki M, Seront E, Dupont S, Hammer J, Legrand C, Brugnara C, Eklund L, Vikkula M, Bischoff J, Boon LM. J Clin Invest. 2015 Sep 1;125(9):3491-504
34. Based on: Ingelman-Sundberg M, 2004 Human drug metabolising cytochrome P450 enzymes: properties and polymorphisms, Naunyn Schmiedebergs Arch Pharmacol. 2004 Jan; 369(1):89-104. And [http://www.medicine.iupui.edu/flockhart/clinlist.htm as of February 15, 2007]

F) Summary of amendment made in 2018 on the initial protocol

Subject : Substantial amendment related to VASE Trial evaluating efficacy of sirolimus in patients with vascular malformations

EudraCT Number : 2015-001703-32

Protocol Number : VASE

Title of Protocol : Phase III multicentric study evaluating the efficacy and safety of sirolimus in Vascular Anomalies that are refractory to standard care.

Study phase : III
Sponsor : Prof. Dr. Laurence Boon

NON-COMMERCIAL TRIAL

Dear Sir/Madam,

Please find enclosed a substantial amendment concerning our Clinical Trial for study drug Rapamune (sirolimus) in patients with complex vascular anomalies that are refractory to standard care such as medical treatment, surgical resection and/or sclerotherapy/embolization (ineffective or accompanied by major complications). This trial is currently ongoing, with a successful enrollement of patients. More than eighty patients have already been enrolled among the 250 planned patients. Due to the impressive preliminary results, we plan to open a new center in Norway.

This is a substantial amendment because a new center had taken part of this study; few changes also occurred into the informed consent, particularly for new reglementation for patient data protection.

Please Find
1A. Protocol modification with addition of a new center (Norway) ; trackchange
1B. Protocole modification with addition of a new center (Norway) ; PDF

2A. ICF adult with new european amendement 2018), mediation service information and contact information of « Autorité Belge de Protection des Données » ; trackchange
2B. ICF adult with new european amendement 2018), mediation service information and contact information of « Autorité Belge de Protection des Données » ; PDF

3A. ICF parents with new european amendement 2018), mediation service information and contact information of « Autorité Belge de Protection des Données » ; trackchange
3B. ICF parents with new european amendement 2018), mediation service information and contact information of « Autorité Belge de Protection des Données » ; PDF

4. Résumé des trackanges

5. Assurance proof

Cover letter Amendement, VASE study, Boon et Seront

1/1

1.
